# Supplementary material for: Inferring fitness landscapes and selection on phenotypic states from single-cell genealogical data
Source: PLoS Genet. 2017 Mar 7;13(3):e1006653. doi: 10.1371/journal.pgen.1006653 (PMC5360348; doi:10.1371/journal.pgen.1006653)
Supplement: S1 Text — (PDF) [file pgen.1006653.s001.pdf]

SUPPORTING INFORMATION

Inferring fitness landscapes and selection on phenotypic states from single-cell genealogical data

Takashi Nozoe, Edo Kussell, Yuichi Wakamoto

Contents

1 Theory **2**
1.1 Definitions of chronological and retrospective probabilities 2
1.2 Definition of fitness landscape 3
1.3 Selection strength 5
1.4 Gaussian approximation and cumulant expansion 7
1.5 Separating individual response and selection in population 10

2 Data analysis **10**
2.1 Notations 11
2.2 Coarse-grained phenotypes assigned to lineages 11
2.3 Coefficient of variation for coarse-grained lineage phenotype 11
2.4 Chronological and retrospective lineage probabilities 12
2.5 Fitness landscape and selection strength 12
2.6 Error estimates for fitness landscape and selection strength 13
2.7 Selection strengths for randomly shuffled phenotype 13
2.8 Calculation of autocorrelation functions of fluorescent intensities 14

3 Application to models **15**
3.1 Markov model for population dynamics with phenotypic fluctuation 15
3.2 Selection strength and derivative of long-term population growth rate 18
3.3 Two-state switching model 20
3.4 Simple stochastic gene expression model 22

4 Materials and Methods **24**
4.1 Plasmids 24
4.2 E. coli strains 24

Supplemental References **26**

1 Theory

1.1 Definitions of chronological and retrospective probabilities

We denote an individual lineage tree originated from a single ancestor cell by $\mathcal{T}$. The time-length of an individual tree $\mathcal{T}$ is fixed and denoted by τ. We suppose that $\mathcal{T}$ is realized with probability $\mathcal{P}(\mathcal{T})$. Let $N(\mathcal{T})$ be the number of cell lineages in a tree $\mathcal{T}$. Individual lineages are labeled by $i = 1, 2, \cdots, N(\mathcal{T})$ as done in Main Text (Fig 2A in Main Text). We denote the number of division events on cell lineage i by D_i, and a phenotype of lineage i by x_i.

For a given tree $\mathcal{T}$, we can count the number of cell lineages with D and x as

$$n\left(D, x|\mathcal{T}\right) \equiv \sum_{i=1}^{N(\mathcal{T})} \delta_{D_i,D}\delta_{x_i,x}, \tag{S1.1}$$

where $\delta_{D_i,D}$ and $\delta_{x_i,x}$ are Kronecker Delta. Then,

$$\sum_{D,x} n\left(D, x|\mathcal{T}\right) = N(\mathcal{T}). \tag{S1.2}$$

We define $P^{\mathrm{cl}}\left(D, x|\mathcal{T}\right)$ as

$$P^{\mathrm{cl}}(D, x|\mathcal{T}) \equiv 2^{-D} n(D, x|\mathcal{T}) \geq 0. \tag{S1.3}$$

We can show that $P^{\mathrm{cl}}(D, x|\mathcal{T})$ is indeed probability distribution as follows: We fix a tree $\mathcal{T}$. Let A_i be an event that one reaches the end point of lineage i descending from the ancestor cell chronologically. At every division (i.e. branch point of lineage trees), one of the two daughter cells is chosen with probability 1/2. Then, $\mathrm{Prob}\left(A_i\right) = 2^{-D_i}$. Since $A_i (i = 1, \cdots N(\mathcal{T}))$ are mutually exclusive,

$$\sum_{i=1}^{N(\mathcal{T})} 2^{-D_i} = \sum_{i=1}^{N(\mathcal{T})} \mathrm{Prob}\left(A_i\right) = \mathrm{Prob}\left(\cup_i A_i\right) = 1. \tag{S1.4}$$

Inserting Eq.S1.1 into Eq.S1.3 and summing $P^{\mathrm{cl}}\left(D, x|\mathcal{T}\right)$ for D and x find

$$\begin{aligned}
\sum_{D,x} P^{\mathrm{cl}}\left(D, x|\mathcal{T}\right) &= \sum_{D,x} 2^{-D} \sum_{i=1}^{N(\mathcal{T})} \delta_{D_i,D}\delta_{x_i,x} \\
&= \sum_{i=1}^{N(\mathcal{T})} \sum_{D} 2^{-D}\delta_{D_i,D} \sum_{x} \delta_{x_i,x} \\
&= \sum_{i=1}^{N(\mathcal{T})} 2^{-D_i} \\
&= 1,
\end{aligned} \tag{S1.5}$$

because $\sum_x \delta_{x_i,x} = 1$ and $\sum_D 2^{-D}\delta_{D_i,D} = 2^{-D_i}$. Therefore, $P^{\mathrm{cl}}(D, x|\mathcal{T})$ is probability distribution.

The factor 2^{-D} in the definition Eq.S1.3 is the probability that a cell lineage on which division occurred D times is chosen when a lineage is selected from an ancestor to a descendant cell chronologically. Because a tree $\mathcal{T}$ contains $n\left(D, x|\mathcal{T}\right)$ lineages whose division count and

phenotype are D and x, the probability that we choose a lineage with D and x in a specific lineage tree $\mathcal{T}$ becomes $2^{-D}n\left(D,x|\mathcal{T}\right)$.

The realization of $\mathcal{T}$ is also probabilistic. Thus, by taking the average of $P^{\rm cl}(D,x|\mathcal{T})$ with respect to $\mathcal{P}(\mathcal{T})$, we can define the *chronological* joint probability distribution of D and x as

$$\begin{aligned} P^{\rm cl}(D,x) &\equiv \sum_{\mathcal{T}} P^{\rm cl}(D,x|\mathcal{T})\mathcal{P}(\mathcal{T}) \\ &= 2^{-D}\sum_{\mathcal{T}} n\left(D,x|\mathcal{T}\right)\mathcal{P}(\mathcal{T}) \\ &= 2^{-D}n(D,x), \end{aligned} \tag{S1.6}$$

where $n(D,x) \equiv \sum_{\mathcal{T}} n\left(D,x|\mathcal{T}\right)\mathcal{P}(\mathcal{T})$ is the expected number of cell lineages with D and x in a tree.

We also define the *retrospective* joint probability of D and x as

$$P^{\rm rs}(D,x) \equiv \frac{n(D,x)}{\langle N(\mathcal{T})\rangle}, \tag{S1.7}$$

where $\langle N(\mathcal{T})\rangle \equiv \sum_{\mathcal{T}} N(\mathcal{T})\mathcal{P}(\mathcal{T})$ is the expected total number of cell lineages in a tree. $\langle N(\mathcal{T})\rangle$ is also expressed as

$$\begin{aligned} \langle N(\mathcal{T})\rangle &= \sum_{\mathcal{T}} \left(\sum_{D,x} n(D,x|\mathcal{T})\right)\mathcal{P}(\mathcal{T}) \\ &= \sum_{D,x} n(D,x) \\ &= \sum_{D,x} 2^{D}P^{\rm cl}(D,x). \end{aligned} \tag{S1.8}$$

We define population growth rate Λ as

$$\Lambda \equiv \frac{1}{\tau}\ln\langle N(\mathcal{T})\rangle = \frac{1}{\tau}\ln\sum_{D,x} 2^{D}P^{\rm cl}(D,x). \tag{S1.9}$$

Eq.S1.7 can be rewritten as

$$P^{\rm rs}(D,x) = e^{-\tau\Lambda}n(D,x). \tag{S1.10}$$

Thus, from Eq.S1.6,

$$P^{\rm rs}(D,x) = P^{\rm cl}(D,x)2^{D}e^{-\tau\Lambda}. \tag{S1.11}$$

1.2 Definition of fitness landscape

We define fitness landscape $h(x)$ so that $e^{\tau h(x)}$ becomes the ratio of the expected number of cell lineages with phenotype x per single lineage tree to the expected chronological probability that we find a cell lineage with x in a tree, i.e.

$$h(x) \equiv \frac{1}{\tau}\ln\frac{\sum_{D} n(D,x)}{\sum_{D} P^{\rm cl}(D,x)}. \tag{S1.12}$$

Let $P^{\rm cl}(x) \equiv \sum_D P^{\rm cl}(D,x)$ and $P^{\rm rs}(x) \equiv \sum_D P^{\rm rs}(D,x)$ be chronological and retrospective marginal probability distributions of phenotype x, respectively. Because $\sum_D n(D,x) = \langle N(\mathcal{T})\rangle P^{\rm rs}(x)$ from Eq.S1.7, $h(x)$ can be rewritten as

$$h(x) = \Lambda + \frac{1}{\tau} \ln \frac{P^{\rm rs}(x)}{P^{\rm cl}(x)}. \tag{S1.13}$$

Thus,

$$P^{\rm rs}(x) = P^{\rm cl}(x) e^{\tau h(x) - \tau\Lambda}. \tag{S1.14}$$

This indicates that when $h(x) > \Lambda$, phenotype x is over-represented in retrospective probability.

In addition, $h(x)$ can be expressed as

$$h(x) = \frac{1}{\tau} \ln \sum_D 2^D P^{\rm cl}(D|x), \tag{S1.15}$$

where $P^{\rm cl}(D|x) = P^{\rm cl}(D,x)/P^{\rm cl}(x) = \frac{2^{-D} n(D,x)}{\sum_D P^{\rm cl}(D,x)}$ is the chronological distribution of D conditioned on x. Eq.S1.15 tells us that $h(x)$ is a specific value of the cumulant generating function of $P^{\rm cl}(D|x)$. Thus, we introduce the time-scaled cumulant generating function of $P^{\rm cl}(D|x)$ as

$$g(\xi|x) \equiv \frac{1}{\tau} \ln \sum_D e^{\xi D} P^{\rm cl}(D|x). \tag{S1.16}$$

Then,

$$h(x) = g(\ln 2|x). \tag{S1.17}$$

$h(x)$ can be expanded with the cumulants of $P^{\rm cl}(D|x)$; if we ignore the cumulants higher than the second-order, we obtain

$$\begin{aligned} \tau h(x) &\approx E\left[D|x\right]^{\rm cl} \ln 2 + \frac{1}{2} Var\left[D|x\right] (\ln 2)^2 \\ &= E\left[D \ln 2|x\right]^{\rm cl} + \frac{1}{2} Var\left[D \ln 2|x\right]^{\rm cl}, \end{aligned} \tag{S1.18}$$

where $E[\cdot|x]^{\rm cl}$ and $Var[\cdot|x]^{\rm cl}$ are mean and variance with respect to chronological conditional probability $P^{\rm cl}(D|x)$.

Furthermore, by taking the averages of Eq.S1.18 with respect to $P^{\rm cl}(x)$,

$$E\left[\tau h(x)\right]^{\rm cl} = E\left[D \ln 2\right]^{\rm cl} + \frac{1}{2} E\left[Var\left[D \ln 2|x\right]^{\rm cl}\right]^{\rm cl}. \tag{S1.19}$$

This equation connects the mean of fitness landscape to the variation of division counts when x is fixed.

Examples

Here we see two extreme cases. When D and x are independent in chronological probability, $P^{\rm cl}(D|x) = P^{\rm cl}(D)$. From Eq.S1.15,

$$\begin{aligned} h(x) &= \frac{1}{\tau} \ln \sum_D 2^D P^{\rm cl}(D) = \frac{1}{\tau} \ln \sum_D \sum_x 2^D P^{\rm cl}(D,x) \\ &= \frac{1}{\tau} \ln \sum_D \sum_x n(D,x) = \frac{1}{\tau} \ln \langle N(\mathcal{T})\rangle \\ &= \Lambda. \end{aligned} \tag{S1.20}$$

Therefore, $h(x)$ always equals to Λ irrespectively of x. From Eq.S1.14, we also obtain $P^{\mathrm{cl}}(x) = P^{\mathrm{rs}}(x)$, which means that when D and x are independent, chronological and retrospective probabilities of x become identical.

In the totally opposite case where D is a deterministic function of x, i.e. $\widetilde{D}(x)$, $P^{\mathrm{cl}}(D,x) = P^{\mathrm{cl}}(x)\delta_{D,\widetilde{D}(x)}$. Thus,

$$\begin{aligned} h(x) &= \frac{1}{\tau}\ln\sum_D 2^D \frac{P^{\mathrm{cl}}(D,x)}{P^{\mathrm{cl}}(x)} \\ &= \frac{1}{\tau}\ln\sum_D 2^D \delta_{D,\widetilde{D}(x)} = \frac{\widetilde{D}(x)}{\tau}\ln 2, \end{aligned} \tag{S1.21}$$

which indicates that the fitness landscape is determined only by $\widetilde{D}(x)$.

1.3 Selection strength

We define selection strength $S[x]$ as

$$\begin{aligned} S\left[x\right] &\equiv \langle h(x)\rangle^{\mathrm{rs}} - \langle h(x)\rangle^{\mathrm{cl}} \\ &= \sum_x \left(\Lambda + \frac{1}{\tau}\ln\frac{P^{\mathrm{rs}}(x)}{P^{\mathrm{cl}}(x)}\right)P^{\mathrm{rs}}(x) \\ &\quad -\sum_x \left(\Lambda + \frac{1}{\tau}\ln\frac{P^{\mathrm{rs}}(x)}{P^{\mathrm{cl}}(x)}\right)P^{\mathrm{cl}}(x) \\ &= \frac{1}{\tau}\sum_x \left(P^{\mathrm{rs}}(x) - P^{\mathrm{cl}}(x)\right)\ln\frac{P^{\mathrm{rs}}(x)}{P^{\mathrm{cl}}(x)} \\ &= \frac{1}{\tau}J[P^{\mathrm{cl}}(x), P^{\mathrm{rs}}(x)], \end{aligned} \tag{S1.22}$$

where $J[P^{\mathrm{cl}}(x), P^{\mathrm{rs}}(x)]$ is Jeffreys divergence. Jeffreys divergence is decomposed into two Kullback-Leibler divergences, i.e.

$$J\left[p(x), q(x)\right] = D_{\mathrm{KL}}\left[p(x)||q(x)\right] + D_{\mathrm{KL}}\left[q(x)||p(x)\right], \tag{S1.23}$$

where

$$D_{\mathrm{KL}}\left[p(x)||q(x)\right] \equiv \sum_x p(x)\ln\frac{p(x)}{q(x)}. \tag{S1.24}$$

Note that

$$\langle h(x)\rangle^{\mathrm{rs}} - \Lambda = \frac{1}{\tau}D_{\mathrm{KL}}\left[P^{\mathrm{rs}}(x)||P^{\mathrm{cl}}(x)\right] \tag{S1.25}$$

and

$$\Lambda - \langle h(x)\rangle^{\mathrm{cl}} = \frac{1}{\tau}D_{\mathrm{KL}}\left[P^{\mathrm{cl}}(x)||P^{\mathrm{rs}}(x)\right] \tag{S1.26}$$

are also candidates of a measure of selection strength. Eq.S1.22, S1.25, and S1.26 are the fundamental relations that link the fitness differences to the statistical deviations between chronological and retrospective probability distributions of x.

Since fitness landscape for D becomes

$$\begin{aligned}\widetilde{h}(D) &= \frac{1}{\tau}\ln\sum_{D'} 2^{D'} P^{\mathrm{cl}}(D'|D) \\ &= \frac{1}{\tau}\ln\sum_{D'} 2^{D'}\delta_{D',D} = \frac{D}{\tau}\ln 2,\end{aligned} \tag{S1.27}$$

the selection strength for division count D is

$$S\left[D\right] = \frac{1}{\tau}\langle D\ln 2\rangle^{\mathrm{rs}} - \frac{1}{\tau}\langle D\ln 2\rangle^{\mathrm{cl}} = \frac{1}{\tau} J\left[P^{\mathrm{cl}}(D), P^{\mathrm{rs}}(D)\right]. \tag{S1.28}$$

As shown below, $S[D]$ imposes the upper bound of selection strength for any phenotype x.

Conditional KL divergence is defined by

$$D_{\mathrm{KL}}\left[p(x|y)||q(x|y)\right] \equiv \sum_{x,y} p(x,y)\ln\frac{p(x|y)}{q(x|y)}. \tag{S1.29}$$

Likewise, conditional Jeffreys divergence is defined as

$$\begin{aligned}J\left[p(x|y), q(x|y)\right] \equiv\ & D_{\mathrm{KL}}\left[p(x|y)||q(x|y)\right] \\ &+ D_{\mathrm{KL}}\left[q(x|y)||p(x|y)\right].\end{aligned} \tag{S1.30}$$

Thus, it is natural to define selection strength of D conditioned on x as

$$S\left[D|x\right] \equiv \frac{1}{\tau} J\left[P^{\mathrm{cl}}(D|x), P^{\mathrm{rs}}(D|x)\right]. \tag{S1.31}$$

The KL divergence for joint probability distributions is decomposed to those for conditional and marginal distributions (chain rule):

$$\begin{aligned}D_{\mathrm{KL}}\left[p(x,y)||q(x,y)\right] =\ & D_{\mathrm{KL}}\left[p(x|y)||q(x|y)\right] \\ &+ D_{\mathrm{KL}}\left[p(y)||q(y)\right].\end{aligned} \tag{S1.32}$$

Evidently, the similar relation holds for Jeffreys divergence:

$$\begin{aligned}J\left[P^{\mathrm{cl}}(D,x), P^{\mathrm{rs}}(D,x)\right] =\ & J\left[P^{\mathrm{cl}}(D|x), P^{\mathrm{rs}}(D|x)\right] \\ &+ J\left[P^{\mathrm{cl}}(x), P^{\mathrm{rs}}(x)\right].\end{aligned} \tag{S1.33}$$

In fact, we can show that

$$J\left[P^{\mathrm{cl}}(D,x), P^{\mathrm{rs}}(D,x)\right] = J\left[P^{\mathrm{cl}}(D), P^{\mathrm{rs}}(D)\right], \tag{S1.34}$$

because

$$\ln\frac{P^{\mathrm{rs}}(D,x)}{P^{\mathrm{cl}}(D,x)} = \ln\frac{\frac{n(D,x)}{\langle N(\mathcal{T})\rangle}}{2^{-D}n(D,x)} = D\ln 2 - \tau\Lambda, \tag{S1.35}$$

and

$$
\begin{aligned}
J\left[P^{\mathrm{cl}}(D,x),P^{\mathrm{rs}}(D,x)\right] &= \sum_{D,x}\Big(P^{\mathrm{rs}}(D,x)-P^{\mathrm{cl}}(D,x)\Big) \\
&\quad \times \ln\frac{P^{\mathrm{rs}}(D,x)}{P^{\mathrm{cl}}(D,x)} \\
&= \sum_{D,x}\Big(P^{\mathrm{rs}}(D,x)-P^{\mathrm{cl}}(D,x)\Big) \\
&\quad \times (D\ln 2-\tau\Lambda) \\
&= \langle D\ln 2\rangle^{\mathrm{rs}}-\langle D\ln 2\rangle^{\mathrm{cl}} \\
&= J[P^{\mathrm{cl}}(D),P^{\mathrm{rs}}(D)]. \qquad \text{(S1.36)}
\end{aligned}
$$

Thus, we can derive from Eq.S1.22, S1.27, and S1.31-S1.34,

$$
S\left[D\right]=S\left[D|x\right]+S\left[x\right]. \qquad \text{(S1.37)}
$$

Evidently, $S\left[D\right]\geq S\left[x\right]$. $S[x]$ reports to what extent variation of x affects the variation of D.

1.4 Gaussian approximation and cumulant expansion

Below we show that selection strength is related to the variance of fitness and also to the fitness correlation between division count and phenotype. We consider the joint probability distribution of $y=\frac{D\ln 2}{\tau}$ and $z=h(x)$. From Eq.S1.15,

$$
e^{\tau h(x)}=\sum_{D}2^{D}P^{\mathrm{cl}}(D|x). \qquad \text{(S1.38)}
$$

Here we put three assumptions:

1. z corresponds to x in a one-to-one manner.
2. y and z can be assumed as continuous variables.
3. $P^{\mathrm{cl}}(y,z)$ follows bivariate normal distribution.

From the first and second assumptions, Eq.S1.38 can be rewritten as

$$
e^{\tau z}=\int e^{\tau y}P^{\mathrm{cl}}(y|z)dy. \qquad \text{(S1.39)}
$$

Further, by using the properties of bivariate normal distributions, we derive the relations among the means and the variances for y and z. When $P^{\mathrm{cl}}(y,z)$ follows bivariate normal distribution, $P^{\mathrm{cl}}(y|z)$ becomes normal distribution, and the right-side of Eq.S1.39 is the moment generating function of $P^{\mathrm{cl}}(y|z)$. Thus,

$$
e^{\tau z}=e^{\tau E[y|z]^{\mathrm{cl}}+\frac{\tau}{2}\alpha^{2}(1-r^{2})}, \qquad \text{(S1.40)}
$$

where

$$
\begin{aligned}
\alpha^{2} &\equiv \tau Var[y]^{\mathrm{cl}} \qquad \text{(S1.41)} \\
r &\equiv \frac{Cov[y,z]^{\mathrm{cl}}}{\sqrt{Var[y]^{\mathrm{cl}}Var[z]^{\mathrm{cl}}}}. \qquad \text{(S1.42)}
\end{aligned}
$$

We used

$$Var[y|x]^{\rm cl} = Var[y]^{\rm cl}(1-r^2) = \frac{\alpha^2(1-r^2)}{\tau} \tag{S1.43}$$

to obtain Eq.S1.40. The logarithm of Eq.S1.40 gives

$$E[y|z]^{\rm cl} = z - \frac{\alpha^2(1-r^2)}{2}. \tag{S1.44}$$

By taking the averages of the both-sides of Eq.S1.44 for $P^{\rm cl}(z)$,

$$E[y]^{\rm cl} = E[z]^{\rm cl} - \frac{\alpha^2(1-r^2)}{2}. \tag{S1.45}$$

Also, by taking the variances,

$$Var[E[y|z]^{\rm cl}]^{\rm cl} = Var[z]^{\rm cl}. \tag{S1.46}$$

Covariance can be written as

$$\begin{aligned} Cov[y,z]^{\rm cl} &= E[yz]^{\rm cl} - E[y]^{\rm cl}E[z]^{\rm cl} \\ &= E[zE[y|z]^{\rm cl}]^{\rm cl} - E[y]^{\rm cl}E[z]^{\rm cl}. \end{aligned} \tag{S1.47}$$

Inserting Eq.S1.44 and S1.45 into Eq.S1.47 finds

$$Cov[y,z]^{\rm cl} = Var[z]^{\rm cl}. \tag{S1.48}$$

Thus, from the definition of r in Eq.S1.42,

$$Var[z]^{\rm cl} = r^2 Var[y]^{\rm cl}. \tag{S1.49}$$

We next consider how the means and the variances of y and z are related between the chronological and the retrospective probabilities. From Eq.S1.11, the chronological and the retrospective probabilities are related to each other by

$$P^{\rm rs}(y,z) = e^{\tau(y-\Lambda)}P^{\rm cl}(y,z). \tag{S1.50}$$

The cumulant generating function of $P^{\rm rs}(y,z)$ is

$$\begin{aligned} C^{\rm rs}(p,q) &\equiv \ln\int_{-\infty}^{\infty}\int_{-\infty}^{\infty} e^{py}e^{qz}P^{\rm rs}(y,z)dydz \\ &= -\Lambda\tau + \ln\int_{-\infty}^{\infty}\int_{-\infty}^{\infty} e^{(p+\tau)y}e^{qz}P^{\rm cl}(y,z)dydz \\ &= -\Lambda\tau + C^{\rm cl}(p+\tau,q) \\ &= -\Lambda\tau + (p+\tau)E[y]^{\rm cl} + qE[z]^{\rm cl} \\ &\quad + \frac{1}{2}\left\{(p+\tau)^2 Var[y]^{\rm cl} + 2(p+\tau)qCov[y,z]^{\rm cl} + q^2 Var[z]^{\rm cl}\right\}. \end{aligned} \tag{S1.51}$$

The above is exact for the Gaussian case, and in general it corresponds to the cumulant expansion to second order. Thus,

$$\begin{aligned}
E[y]^{\rm rs} &= \frac{\partial C^{\rm rs}(p,q)}{\partial p}|_{p=q=0} \\
&= E[y]^{\rm cl} + \tau Var[y]^{\rm cl}, && \text{(S1.52a)} \\
E[z]^{\rm rs} &= \frac{\partial C^{\rm rs}(p,q)}{\partial q}|_{p=q=0} = E[z]^{\rm cl} + \tau Cov[y,z]^{\rm cl} \\
&= E[z]^{\rm cl} + \tau Var[z]^{\rm cl}, && \text{(S1.52b)} \\
Var[y]^{\rm rs} &= \frac{\partial^2 C^{\rm rs}(p,q)}{\partial p^2}|_{p=q=0} = Var[y]^{\rm cl}, && \text{(S1.52c)} \\
Var[z]^{\rm rs} &= \frac{\partial^2 C^{\rm rs}(p,q)}{\partial q^2}|_{p=q=0} = Var[z]^{\rm cl}, && \text{(S1.52d)} \\
Cov[y,z]^{\rm rs} &= \frac{\partial^2 C^{\rm rs}(p,q)}{\partial p \partial q}|_{p=q=0} = Cov[y,z]^{\rm cl}. && \text{(S1.52e)}
\end{aligned}$$

From the definition of selection strength (Eq.S1.22 and S1.28),

$$\begin{aligned}
S[D] &= E[y]^{\rm rs} - E[y]^{\rm cl} = \tau Var[y]^{\rm cl}, && \text{(S1.53)} \\
S[x] &= E[z]^{\rm rs} - E[z]^{\rm cl} = \tau Var[z]^{\rm cl}. && \text{(S1.54)}
\end{aligned}$$

Therefore, selection strength (and equivalently fitness difference between retrospective and chronological distributions) is linked with the fitness variance of the corresponding phenotype. Furthermore,

$$S_{\rm rel}[x] \equiv \frac{S[x]}{S[D]} = \frac{Var[z]^{\rm cl}}{Var[y]^{\rm cl}} = r^2, \quad \text{(S1.55)}$$

from Eq.S1.49, which shows that selection strength is also related to the fitness correlation between division count and phenotype.

In addition,

$$\begin{aligned}
S[D|x] &= S[D] - S[x] \\
&= \tau(Var[y]^{\rm cl} - Var[z]^{\rm cl}) \\
&= \tau(1-r^2)Var[y]^{\rm cl} \\
&= (1-r^2)\alpha^2, && \text{(S1.56)}
\end{aligned}$$

from Eq.S1.37, S1.41, S1.49, S1.53, S1.54. Since $E[Var[y|x]^{\rm cl}]^{\rm cl} = E\left[\frac{\alpha^2(1-r^2)}{\tau}\right]^{\rm cl} = \frac{\alpha^2(1-r^2)}{\tau}$ from Eq.S1.43,

$$S[D|x] = \tau E[Var[y|x]^{\rm cl}]^{\rm cl}. \quad \text{(S1.57)}$$

$S[x]$ is also rewritten as

$$S[x] = \tau Var[z]^{\rm cl} = \tau Var[E[y|z]^{\rm cl}]^{\rm cl} \quad \text{(S1.58)}$$

from Eq.S1.54 and S1.46. Thus, the relationship of selection strength Eq.S1.37 corresponds to the well-known *law of total variance*

$$Var[y]^{\rm cl} = E[Var[y|z]^{\rm cl}]^{\rm cl} + Var[E[y|z]^{\rm cl}]^{\rm cl}. \quad \text{(S1.59)}$$

The term $Var[E[y|z]^{\rm cl}]^{\rm cl}$ on the right-side is referred to as "informational component" of parameter z for the total variance of y [1]. Thus, in our context, $S[x]$ can be regarded as the informational component of x for the total fitness variation.

1.5 Separating individual response and selection in population

We next describe the general framework for distinguishing selection in population from individual response. For this purpose, we introduce environmental parameter $\mathcal{E}$ and denote chronological probability distribution by $P^{\mathrm{cl}}(D, x|\mathcal{E})$.

Then, fitness landscape and population growth rate are re-defined as

$$h(x;\mathcal{E}) \equiv \frac{1}{\tau}\ln\sum_{D} 2^D P^{\mathrm{cl}}(D|x,\mathcal{E}), \tag{S1.60}$$

and

$$\Lambda(\mathcal{E}) \equiv \frac{1}{\tau}\ln\sum_{D,x} 2^D P^{\mathrm{cl}}(D,x|\mathcal{E}), \tag{S1.61}$$

from Eq.S1.9 and S1.15. The retrospective probability distribution is

$$P^{\mathrm{rs}}(D,x|\mathcal{E}) \equiv 2^D e^{-\tau\Lambda(\mathcal{E})} P^{\mathrm{cl}}(D,x|\mathcal{E}), \tag{S1.62}$$

from Eq.S1.11. For marginal distributions of x,

$$P^{\mathrm{rs}}(x|\mathcal{E}) = e^{\tau h(x;\mathcal{E})-\tau\Lambda(\mathcal{E})} P^{\mathrm{cl}}(x|\mathcal{E}), \tag{S1.63}$$

from Eq.S1.14. Note that $\mathcal{E}$ is a normal parameter, not a random variable. We add $\mathcal{E}$ to the upper index of the expectation to explicitly indicate the dependence of the probability distributions on $\mathcal{E}$. Selection strengths are defined as

$$S_{\mathcal{E}}[D] \equiv \frac{1}{\tau}E[D\ln 2]^{\mathrm{rs},\mathcal{E}} - \frac{1}{\tau}E[D\ln 2]^{\mathrm{cl},\mathcal{E}}, \tag{S1.64}$$

and

$$S_{\mathcal{E}}[x] \equiv E[h(x;\mathcal{E})]^{\mathrm{rs},\mathcal{E}} - E[h(x;\mathcal{E})]^{\mathrm{cl},\mathcal{E}}. \tag{S1.65}$$

Eq.S1.65 should be rewritten as

$$E[h(x;\mathcal{E})]^{\mathrm{rs},\mathcal{E}} = E[h(x;\mathcal{E})]^{\mathrm{cl},\mathcal{E}} + S_{\mathcal{E}}[x]. \tag{S1.66}$$

The left-side of Eq.S1.66 represents the mean fitness for $P^{\mathrm{rs}}(x|\mathcal{E})$, and the right side is composed of two terms: the mean fitness for $P^{\mathrm{cl}}(x|\mathcal{E})$, and the selection strength in the environment $\mathcal{E}$. Thus, one can express the change of retrospective mean fitness as the sum of the two terms: one representing the change of chronological mean fitness (individual response), and the other representing the change of selection strength:

$$E[h(x;\mathcal{E})]^{\mathrm{rs},\mathcal{E}'} - E[h(x;\mathcal{E})]^{\mathrm{rs},\mathcal{E}} = (E[h(x;\mathcal{E})]^{\mathrm{cl},\mathcal{E}'} - E[h(x;\mathcal{E})]^{\mathrm{cl},\mathcal{E}}) + (S_{\mathcal{E}'}[x] - S_{\mathcal{E}}[x]). \tag{S1.67}$$

We are therefore able to separately evaluate the contribution from individual response and the one from selection strength by calculating and comparing $E[h(x;\mathcal{E})]^{\mathrm{cl},\mathcal{E}'} - E[h(x;\mathcal{E})]^{\mathrm{cl},\mathcal{E}}$ and $S_{\mathcal{E}'}[x] - S_{\mathcal{E}}[x]$.

2 Data analysis

Here we describe how we evaluated fitness landscape and selection strength from the single-cell lineage tree data.

2.1 Notations

We denote the time-lapse interval by Δt (= 5 min). We define the discrete time steps as $t_k = k\Delta t$ for $k = 0, 1, \cdots, E$, where $t_E = E\Delta t$ is the end time. We label individual trees in a dataset by $j = 1, \cdots, M$, where M denotes the number of cells at the start time, and we label members of tree 1 by $i = 1, \cdots, N_1$, those of tree 2 by $i = N_1 + 1, \cdots, N_1 + N_2$, and so on, where N_j denotes the number of lineages in tree j. Thus, the total number of cells at t_E is $N_{\mathrm{tot}} = \sum_{j=1}^{M} N_j$. We introduce a function φ that returns the label of tree to which a lineage belongs; when lineage i is a member of tree j, $\varphi(i) = j$. That is, $\varphi(i) = j$ if $N_{j-1} + 1 \leq i \leq N_j$ (we define $N_0 = 0$).

From single-cell time-lapse experiments, we can obtain the information on the transitions of individual cells' phenotypes. Let the cell volume and protein concentration of a cell at time t on lineage i be $v_i(t)$ and $c_i(t)$, respectively. In the analysis, we approximated $v_i(t)$ by cell area projected on a two-dimensional image, and $c_i(t)$ by mean fluorescent intensity within the corresponding cell area. We denote the entire time-courses of cellular volume and protein concentration by $\boldsymbol{v}_i \equiv \{v_i(t_k)\}_{0\leq k\leq E}$ and $\boldsymbol{c}_i \equiv \{c_i(t_k)\}_{0\leq k\leq E}$, respectively. We also introduce the time-series of cell divisions: We define $b_i(t_k)$ by $b_i(t_k) = 1$ if division occurred at time t_{k+1} and otherwise $b_i(t_k) = 0$. We define $\boldsymbol{b}_i \equiv \{b_i(t_k)\}_{0\leq k\leq E-1}$.

2.2 Coarse-grained phenotypes assigned to lineages

We calculated the coarse-grained phenotypes described in Main Text by

$$D_i \equiv \sum_{k=0}^{E-1} b_i(t_k), \tag{S2.1}$$

$$\overline{c}_i \equiv \frac{1}{E+1} \sum_{k=0}^{E} c_i(t_k), \tag{S2.2}$$

$$\overline{\lambda}_i \equiv \frac{\sum_{k=0}^{E-1} (1 - b_i(t_k)) \ln \frac{v_i(t_{k+1})}{v_i(t_k)}}{\sum_{k=0}^{E-1} (1 - b_i(t_k))}, \tag{S2.3}$$

and

$$\overline{p}_i \equiv \frac{\sum_{k=0}^{E-1} (1 - b_i(t_k)) \left(c_i(t_{k+1}) - c_i(t_k) + c_i(t_k) \ln \frac{v_i(t_{k+1})}{v_i(t_k)} \right)}{\sum_{k=0}^{E-1} (1 - b_i(t_k))}. \tag{S2.4}$$

D_i is the number of cell divisions of lineage i; $\overline{c}_i$ is the time-averaged protein concentration of lineage i; $\overline{\lambda}_i$ is the time-averaged elongation rate of lineage i; and $\overline{p}_i$ is the time-averaged protein production rate of lineage i. The denominator of Eq.S2.3 and Eq.S2.4 is $E - D_i$.

2.3 Coefficient of variation for coarse-grained lineage phenotype

We consider one-dimensional and continuous lineage phenotype $\hat{x}_i$ (suppose $\overline{c}_i$, $\overline{p}_i$ and $\overline{\lambda}_i$ as $\hat{x}_i$). Let $P_i^{\mathrm{rs}} = 1/N_{\mathrm{tot}}$ and $P_i^{\mathrm{cl}} = 2^{-D_i}/M$. The coefficient of variation (CV) with chronological and retrospective weights are

$$\mathrm{CV}^{\mathrm{cl}} \equiv \frac{\sqrt{\sum_{i=1}^{N_{\mathrm{tot}}} P_i^{\mathrm{cl}} \hat{x}_i^2 - \left(\sum_{i=1}^{N_{\mathrm{tot}}} P_i^{\mathrm{cl}} \hat{x}_i \right)^2}}{\sum_{i=1}^{N_{\mathrm{tot}}} P_i^{\mathrm{cl}} \hat{x}_i}, \tag{S2.5}$$

and

$$\mathrm{CV}^{\mathrm{rs}} \equiv \frac{\sqrt{\sum_{i=1}^{N_{\mathrm{tot}}} P_i^{\mathrm{rs}} \hat{x}_i^2 - \left(\sum_{i=1}^{N_{\mathrm{tot}}} P_i^{\mathrm{rs}} \hat{x}_i\right)^2}}{\sum_{i=1}^{N_{\mathrm{tot}}} P_i^{\mathrm{rs}} \hat{x}_i}. \tag{S2.6}$$

We calculated chronological and retrospective CVs for lineage phenotypes which are shown in S9 Fig.

2.4 Chronological and retrospective lineage probabilities

We define the interval $I_{x,\Delta x} \equiv \left[x - \frac{\Delta x}{2}, x + \frac{\Delta x}{2}\right)$. and then we can define the chronological and retrospective probability densities for x.

The expectation with respect to $\mathcal{P}(\mathcal{T})$ in Eq.S1.6 is replaced by the arithmetic mean over trees and thereby we define the chronological probability density of x by

$$\begin{aligned}\hat{P}^{\mathrm{cl}}(x) &\equiv \frac{1}{\Delta x}\frac{1}{M}\sum_{j=1}^{M}\sum_{i:\hat{x}_i \in I_{x,\Delta x}, \varphi(i)=j} 2^{-D_i} \\ &= \frac{1}{\Delta x}\frac{1}{M}\sum_{i:\hat{x}_i \in I_{x,\Delta x}} 2^{-D_i}.\end{aligned} \tag{S2.7}$$

Similarly, the expected total number of cell lineages in a tree $\langle N(\mathcal{T})\rangle$ in Eq.S1.7 is replaced by

$$\frac{1}{M}\sum_{j=1}^{M} N_j = \frac{N_{\mathrm{tot}}}{M}. \tag{S2.8}$$

Then, we define the retrospective probability density of x by

$$\begin{aligned}\hat{P}^{\mathrm{rs}}(x) &\equiv \frac{1}{\Delta x}\frac{M}{N_{\mathrm{tot}}}\frac{1}{M}\sum_{j=1}^{M}\sum_{i:\hat{x}_i \in I_{x,\Delta x}, \varphi(i)=j} 1 \\ &= \frac{1}{\Delta x}\frac{1}{N_{\mathrm{tot}}}\sum_{i:\hat{x}_i \in I_{x,\Delta x}} 1.\end{aligned} \tag{S2.9}$$

Here we simply estimate the probability densities by the frequency polygons. That is, x takes discrete values $x_{\min}, x_{\min}+\Delta x, \cdots, x_{\min}+(L-1)\Delta x$, where $x_{\min}$ is the minimum value among $\hat{x}_i$ and L is the number of total bins determined by $L = \lfloor \frac{x_{\max}-x_{\min}}{\Delta x} \rfloor + 2$ where $x_{\max}$ denotes the maximum value among $\hat{x}_i$. We determine Δx from the interquartile range of $\hat{\boldsymbol{x}}$, where $\hat{\boldsymbol{x}} = \{\hat{x}_i\}_{1 \le i \le N_{\mathrm{tot}}}$. Let the interquartile range of $\hat{\boldsymbol{x}}$ denoted by $\mathrm{IQR}(\hat{\boldsymbol{x}})$. Interquartile range is expected to be more robust for outliers than standard deviation; we therefore determined Δx with a constant α as

$$\Delta x = \alpha \cdot \mathrm{IQR}(\hat{\boldsymbol{x}}). \tag{S2.10}$$

As explained before, we choose α so that selection strength of x becomes the least sensitive to the change of Δx.

2.5 Fitness landscape and selection strength

Let $\tau = E\Delta t$. From Eq.S2.8 and Eq.S1.9, the population growth rate $\hat{\Lambda}$ can be evaluated by

$$\hat{\Lambda} \equiv \frac{1}{\tau}\ln\frac{N_{\mathrm{tot}}}{M}. \tag{S2.11}$$

We evaluated fitness landscape $\hat{h}(x)$ by

$$\hat{h}(x) \equiv \frac{1}{\tau} \ln \frac{N_{\mathrm{tot}} P^{\mathrm{rs}}(x)}{M P^{\mathrm{cl}}(x)}, \tag{S2.12}$$

which corresponds to Eq.S1.13 in Sec.1.2. We also evaluated selection strength by

$$\hat{S}_X\left(\Delta x\right) \equiv \frac{1}{\tau} \sum_{l=0}^{L-1} \hat{h}(x_{\min} + l\Delta x) \left(P^{\mathrm{rs}}(x_{\min} + l\Delta x) - P^{\mathrm{cl}}(x_{\min} + l\Delta x) \right) \Delta x, \tag{S2.13}$$

which corresponds to Eq.S1.22 in Sec.1.3. Here we represented selection strength as a function of bin width Δx. We can define selection strength for division count by

$$\hat{S}_D \equiv \frac{1}{\tau} \sum_{i=1}^{N_{\mathrm{tot}}} \left(P_i^{\mathrm{rs}} - P_i^{\mathrm{cl}} \right) D_i \ln 2. \tag{S2.14}$$

where $P_i^{\mathrm{rs}} = 1/N_{\mathrm{tot}}$ and $P_i^{\mathrm{cl}} = 2^{-D_i}/M$. The value of selection strength $\hat{S}_X(\Delta x)$ depends on the bin width Δx. In fact, we can show that $\hat{S}_X(\Delta x) = \hat{S}_D$ if $\Delta x < \Delta \hat{x}_{\min} \equiv \min_{i \neq j} |\hat{x}_i - \hat{x}_j|$, and that $\hat{S}_X(\Delta x) = 0$ if $\Delta x > 2\Delta \hat{x}_{\max} \equiv 2 \max_{i \neq j} |\hat{x}_i - \hat{x}_j|$. Thus, $\hat{S}_X(\Delta x)$ increases from 0 to $\hat{S}_D$ as Δx decreases from $2\Delta \hat{x}_{\max}$ to $\Delta \hat{x}_{\min}$. At the proper level of Δx, we expect that $\hat{S}_X(\Delta x)$ is robust against the change of Δx and shows a plateau, which we indeed found in our experimental data in Fig 3 and 4.

2.6 Error estimates for fitness landscape and selection strength

To evaluate the error ranges of fitness landscapes and selection strengths in the simulation, we calculated mean and standard deviation of 10 numerical simulations for the each condition with different r_g and Hill coefficient n For experimental data analysis, we calculated mean and standard deviation of fitness landscapes and selection strengths of the three independent time-lapse experiments for the each condition of +Sm and −Sm.

In the simulation, we obtained $x_{\min}$ and $x_{\max}$ for each condition of r_g and Hill coefficient n from the 10 datasets and determined the bin width as $0.3\mathrm{IQR}(\hat{\boldsymbol{x}})$.

In the experiment for F3/pTN001, we performed the lineage analysis separately for the early term (from 0 min to 200 min) and the late term (from 200 min to 400 min). In the experiment for F3NW, we performed the lineage analysis for lineages starting from 100 min lasting to 300 min. To assign the same bins for the replicate experiments, we obtained $x_{\min}$ and $x_{\max}$ from the six datasets for F3/pTN001 and eight datasets for F3NW, and determined the bin width as $0.4\mathrm{IQR}(\hat{\boldsymbol{x}})$ for the early term of F3/pTN001, and $0.3\mathrm{IQR}(\hat{\boldsymbol{x}})$ for F3NW and the late term of F3/pTN001. The number of cells at different time points are summarized in S1 Table and S2 Table. In S6 Fig, we show the fitness landscapes and selection strengths for the early term of F3/pTN001; we did not detect the differences of the selection strengths between +Sm and −Sm conditions for all the phenotypes during the early term.

2.7 Selection strengths for randomly shuffled phenotype

To test whether a selection strengths of phenotype x, $S[x]$, reports statistically significant correlation between x and D, we computed selection strengths for randomly shuffled combination of D and x across lineages. Let $\boldsymbol{p} \equiv (p_i)_{i=1,\cdots,N_{\mathrm{tot}}}$ a random permutation of $(1, 2, \cdots, N_{\mathrm{tot}})$,

i.e. $\boldsymbol{p}$ is a realization randomly chosen from all possible $N_{\mathrm{tot}}!$ permutations with equal probability. For a set of $(D_i, \hat{x}_i)$ over $i = 1, 2, \cdots, N_{\mathrm{tot}}$, we randomly shuffle $\hat{x}_i$ and do not shuffle D_i, obtaining a new set $\{(D_i, \hat{x}'_i)\}_{i=1,\cdots,N_{\mathrm{tot}}}$ where $\hat{x}'_i = x_{p_i}$. For the set $\{(D_i, \hat{x}'_i)\}_{i=1,\cdots,N_{\mathrm{tot}}}$, we calculate the selection strength of x as described in Sec.2.4 and Sec.2.5, denoted by $S_{\mathrm{shuffled}}[x]$. $\hat{x}_i$ takes the values D_i, $\overline{\lambda}_i$, $\overline{p}_i$ and $\overline{c}_i$. When $\hat{x}_i$ takes the values $\overline{\lambda}_i$, $\overline{p}_i$ and $\overline{c}_i$, $S_{\mathrm{shuffled}}[x]$ is directly calculated by Eq.S2.13. For D_i, we computed $S_{\mathrm{shuffled}}[D]$ as follows.

We fix a random permutation $\boldsymbol{p}$ and we introduce a new variable $\widetilde{D}_i = D_{p_i}$ as a phenotypic value of the i-th lineage. The i-th lineage divides D_i times while we assume that it has a phenotypic value $\widetilde{D}_i = D_{p_i}$. Then, we can define retrospective and chronological probabilities of $\widetilde{D} = d$ as below:

$$\hat{P}^{\mathrm{cl}}\left(\widetilde{D} = d\right) \equiv \frac{1}{M} \sum_{i:D_{p_i}=d} 2^{-D_i}, \tag{S2.15}$$

$$\hat{P}^{\mathrm{rs}}\left(\widetilde{D} = d\right) \equiv \frac{1}{N_{\mathrm{tot}}} \sum_{i:D_{p_i}=d} 1. \tag{S2.16}$$

Finally we can calculate

$$S_{\mathrm{shuffled}}[D] = \frac{1}{\tau} \sum_d \left(\hat{P}^{\mathrm{rs}}\left(\widetilde{D} = d\right) - \hat{P}^{\mathrm{cl}}\left(\widetilde{D} = d\right)\right) \ln \frac{\hat{P}^{\mathrm{rs}}\left(\widetilde{D} = d\right)}{\hat{P}^{\mathrm{cl}}\left(\widetilde{D} = d\right)}. \tag{S2.17}$$

Since the fitness landscape of $\widetilde{D}$ is given by

$$\hat{h}(\widetilde{D}) = \frac{1}{\tau} \ln \frac{\hat{P}^{\mathrm{rs}}\left(\widetilde{D}\right)}{\hat{P}^{\mathrm{cl}}\left(\widetilde{D}\right)} + \hat{\Lambda} \tag{S2.18}$$

($\hat{\Lambda}$ is defined in Eq.S2.11), Eq.S2.18 is also written as

$$S_{\mathrm{shuffled}}[D] = \sum_d \left(\hat{P}^{\mathrm{rs}}\left(\widetilde{D} = d\right) - \hat{P}^{\mathrm{cl}}\left(\widetilde{D} = d\right)\right) \hat{h}(\widetilde{D} = d). \tag{S2.19}$$

By repeating many times such random shuffling of phenotype and computing $S_{\mathrm{shuffled}}[x]$, we obtain the median and confidence interval. We repeated shuffling 10000 times and computed the 95% CI as shown in S10 Fig.

2.8 Calculation of autocorrelation functions of fluorescent intensities

To calculate the autocorrelation functions, we rewrite the notations for lineages by explicitly denoting different time windows. Similarly to the previous section, the start time and the end time of the whole trees are denoted by $t_0 = 0$ and $t_E = E\Delta t$. The partial time-courses of fluorescent protein concentration are denoted by $\boldsymbol{c}_i^{[l,m]} \equiv \{c_i^{[l,m]}(t_k)\}_{l-m \leq k \leq l}$. m denotes the time-length of the partial timeseries and l denotes the end timepoint of the partial timeseries. Let $N(t_l)$ be the cell number at time t_l and $i = 1, 2, \cdots, N(t_l)$. The set of (i, l, m) uniquely indentifies a partial cell lineage and let the number of divisions of the lineages labled with (i, l, m) denoted by $D_i^{l,m}$.

We define the autocorrelation function of the fluorescent protein concentration weighed with arbitrary weight $w_i^{[l,m]}$ by

$$\Phi(m\Delta t) \equiv \sum_{l=m}^{E}\sum_{i=1}^{N(t_l)} \frac{w_i^{[l,m]}}{W_m} c_i^{[l,m]}(t_{l-m}) c_i^{[l,m]}(t_l) - \sum_{l=m}^{E}\sum_{i=1}^{N(t_l)} \frac{w_i^{[l,m]}}{W_m} c_i^{[l,m]}(t_{l-m}) \sum_{l=m}^{E}\sum_{i=1}^{N(t_l)} \frac{w_i^{[l,m]}}{W_m} c_i^{[l,m]}(t_l) \tag{S2.20}$$

where

$$W_m = \sum_{l=m}^{E}\sum_{i=1}^{N(t_l)} w_i^{[l,m]}. \tag{S2.21}$$

We normalize the autocorrelation function by the square root of the product of

$$V_m \equiv \frac{1}{W_m}\sum_{l=m}^{E}\sum_{i=1}^{N(t_l)} w_i^{[l,m]} c_i^{[l,m]}(t_l)^2 - \left(\frac{1}{W_m}\sum_{l=m}^{E}\sum_{i=1}^{N(t_l)} w_i^{[l,m]} c_i^{[l,m]}(t_l)\right)^2 \tag{S2.22}$$

and

$$\widetilde{V}_m \equiv \frac{1}{W_m}\sum_{l=m}^{E}\sum_{i=1}^{N(t_l)} w_i^{[l,m]} c_i^{[l,m]}(t_{l-m})^2 - \left(\frac{1}{W_m}\sum_{l=m}^{E}\sum_{i=1}^{N(t_l)} w_i^{[l,m]} c_i^{[l,m]}(t_{l-m})\right)^2. \tag{S2.23}$$

V_m is the variance of fluorescent protein concentrations at the end timepoint among the lineages whose time-length is $m\Delta t$, and $\widetilde{V}_m$ is the variance at the start timepoint. That is, the normalized autocorrelation function is gven by

$$\phi(m\Delta t) \equiv \frac{\Phi(m\Delta t)}{\sqrt{V_m \widetilde{V}_m}}. \tag{S2.24}$$

We calculated the normalized autocorrelation function $\phi(m\Delta t)$ for chronological and retrospective weights: $w_i^{[l,m]} = 2^{-D_i^{[l,m]}}$ for chronological and $w_i^{[l,m]} = 1$ for retrospective. We checked the normalized autocorrelation functions with chronological weighing were succesfully consisted to the rigorous function of autocorrelation in the simulation, while the retrospectively weighed autocorrelation functions were more biased as stronger selection acted on the simulated phenotypic state. Normalized autocorrelation functions of the experimental data described in Main Text are shown in S1 Fig.

3 Application to models

We here apply our framework to several models and derive the expressions for fitness landscape and selection strength.

3.1 Markov model for population dynamics with phenotypic fluctuation

We model population dynamics with a large number of cells whose phenotypic traits temporally fluctuate as discrete-state continuous-time Markov process. This model has been studied previously [2, 3]; here we apply our framework to show how our measures of fitness landscape and selection strength are given in this well-studied model.

Let cellular phenotype denoted by x, which may be a multivariate vector in general: x represents a set of values of multiple phenotypic traits (e.g. a set of expression levels of gene A, gene B,... and gene Z). Let the number of cells with phenotype x at time t denoted by $\hat{Y}_t(x)$. Let the environmental conditions denoted by $\mathcal{E}(t)$ that may temporally vary (we assume $\mathcal{E}(t)$ also takes discrete states). Within an infinitesimal time period dt, a cell with phenotype x switches to phenotype x' with probability $s^{\mathcal{E}}(x \to x')dt$ and divides into two identical siblings with probability $f(x)dt$. We define $s^{\mathcal{E}}(x \to x) = 0$. We call $s^{\mathcal{E}}(x \to x')$ the switching rate from x to x' and $f^{\mathcal{E}}(x)$ the instantaneous fitness (reproduction rate) of a cell with phenotype x. The upper index $\mathcal{E}$ indicates that switching rate and instantaneous fitness depend on the current environmental conditions $\mathcal{E}$. We denote the expected number of cells with a phenotype x at time t by $\overline{n}(x,t) \equiv E\left[\hat{Y}_t(x)\right]$, where $E\left[\hat{X}\right]$ represents the expected value of the random variable $\hat{X}$. Then, we can derive the equation for time-evolution of $\overline{n}(x,t)$ as

$$\frac{d}{dt}\overline{n}(x,t) = \left(f^{\mathcal{E}(t)}(x) - \eta^{\mathcal{E}(t)}(x)\right)\overline{n}(x,t) + \sum_{x'} \overline{n}(x',t)s^{\mathcal{E}(t)}(x' \to x), \tag{S3.1}$$

where $\eta^{\mathcal{E}}(x)$ is defined by

$$\eta^{\mathcal{E}}(x) \equiv \sum_{x'} s^{\mathcal{E}}(x \to x'), \tag{S3.2}$$

which is called the escape rate. The summation is taken over the whole set of phenotypes. We define the diagonal matrix $F(t)$ with elements $f^{\mathcal{E}}(x)\delta_{x,x'}$ and the square matrix $S(t)$ with elements $s^{\mathcal{E}}(x' \to x) - \eta^{\mathcal{E}}(x)\delta_{x,x'}$. Then, the solution of Eq.S3.1 can be represented by the summation of the biased path probabilities as shown below.

To obtain this representation, we introduce history formulation of the population dynamics for Eq.S3.1 as done in [3]. We fix a time period τ. Let a phenotype at time t denoted by $x(t)$ and its time-series $\{x(t)\}_{0\leq t\leq \tau}$ by σ. We call σ a phenotypic history. We begin by introducing *chronological path probabilities* of phenotypic history σ, $P^{\mathrm{cl}}(\sigma)$. $P^{\mathrm{cl}}(\sigma)$ is the path probability of σ generated by the switching matrix S. We fix the initial condition of Eq.S3.1 to $\overline{n}(x,0)$ that satisfies $\sum_x \overline{n}(x,0) = 1$ and thereby $\overline{n}(x,0)$ is a probability distribution describing the frequency of phenotype in the initial population. Then, $P^{\mathrm{cl}}(\sigma)$ can be represented as below:

$$P^{\mathrm{cl}}(\sigma) = \lim_{m\to\infty}\left[\prod_{j=0}^{m-1}\left(e^{S\Delta t}\right)_{x((j+1)\Delta t),x(j\Delta t)}\right]\overline{n}(x(0),0) \tag{S3.3}$$

where $\Delta t = \tau/m$. Next, we define *historical fitness*, $H(\sigma)$, as

$$H(\sigma) \equiv \int_0^{\tau} f^{\mathcal{E}(t)}(x(t))dt. \tag{S3.4}$$

Historical fitness $H(\sigma)$ is the time-integral of instantaneous fitness over the entire phenotypic history. In fact, $P^{\mathrm{cl}}(\sigma)e^{H(\sigma)}$ represents the expected number of cells with phenotypic history σ in population growing with instantaneous fitness $f(x)$ [3]. Thus the expected number of cells with phenotype x in the final population (i.e. at time τ) is the sum of $P^{\mathrm{cl}}(\sigma)e^{H(\sigma)}$ over all histories that end in the phenotype x:

$$\overline{n}(x,\tau) = \sum_{\sigma\in\mathcal{H}_{x,\tau}} P^{\mathrm{cl}}(\sigma)e^{H(\sigma)}, \tag{S3.5}$$

where $\mathcal{H}_{x,\tau}$ denotes the space of all phenotypic histories of length τ that end in the phenotype x. This representation gives the solution of Eq.S3.1. Similarly, the expected number of cell in the final population (at time τ) is the sum of $P^{\mathrm{cl}}(\sigma)e^{H(\sigma)}$ over all histories:

$$\overline{N}(\tau) = \sum_{\sigma\in\mathcal{H}_\tau} P^{\mathrm{cl}}(\sigma)e^{H(\sigma)}, \tag{S3.6}$$

where $\mathcal{H}_\tau$ denotes the space of all phenotypic histories of length τ. *Retrospective path probabilities* becomes

$$P^{\mathrm{rs}}(\sigma) = \frac{P^{\mathrm{cl}}(\sigma)e^{H(\sigma)}}{\overline{N}(\tau)}. \tag{S3.7}$$

$P^{\mathrm{rs}}(\sigma)$ represents the frequency of phenotypic history σ in population. In the model described here, in which cells divide depending only on instantaneous fitness $f^{\mathcal{E}}(x)$, cellular division on history σ follows inhomogeneous Poisson process. Consequently, the number of divisions D conditioned on chronological history σ follows Poisson distribution whose mean division number is historical fitness $H(\sigma)$:

$$P^{\mathrm{cl}}(D|\sigma) = \frac{H(\sigma)^D}{D!}e^{-H(\sigma)}. \tag{S3.8}$$

Then, fitness landscape for history σ satisfies

$$e^{\tau h(\sigma)} = \sum_{D=0}^{\infty} 2^D \frac{H(\sigma)^D}{D!}e^{-H(\sigma)} = e^{H(\sigma)}. \tag{S3.9}$$

Thus

$$h(\sigma) = \frac{H(\sigma)}{\tau}. \tag{S3.10}$$

This result shows that fitness landscape for σ is in fact equivalent of historical fitness divided by time length τ in this model.

We can also derive a general consequence for the selection strength. $P^{\mathrm{rs}}(D|\sigma)$ also follows the Poisson distribution because

$$P^{\mathrm{rs}}(D|\sigma) = 2^D e^{-H(\sigma)} P^{\mathrm{cl}}(D|\sigma) = \frac{(2H(\sigma))^D}{D!}e^{-2H(\sigma)}. \tag{S3.11}$$

Thus, the mean division count is $E\left[D|\sigma\right]^{\mathrm{rs}} = 2H(\sigma)$. From Eq.S1.28, the maximum selection strength is

$$\begin{aligned}
S\left[D\right] &= \frac{1}{\tau}\left(E\left[D\right]^{\mathrm{rs}} - E\left[D\right]^{\mathrm{cl}}\right)\ln 2 \\
&= \frac{1}{\tau}\left(E\left[E\left[D|\sigma\right]^{\mathrm{rs}}\right]^{\mathrm{rs}} - E\left[E\left[D|\sigma\right]^{\mathrm{cl}}\right]^{\mathrm{cl}}\right)\ln 2 \\
&= \frac{1}{\tau}\left(2E\left[H(\sigma)\right]^{\mathrm{rs}} - E\left[H(\sigma)\right]^{\mathrm{cl}}\right)\ln 2 \\
&= \left(2S\left[\sigma\right] + E\left[h(\sigma)\right]^{\mathrm{cl}}\right)\ln 2.
\end{aligned} \tag{S3.12}$$

In the third row, we used $E\left[D|\sigma\right]^{\mathrm{rs}} = 2H(\sigma)$ and $E\left[D|\sigma\right]^{\mathrm{cl}} = H(\sigma)$; in the last row, we used $h(\sigma) = H(\sigma)/\tau$ and $S\left[\sigma\right] = E\left[h(\sigma)\right]^{\mathrm{rs}} - E\left[h(\sigma)\right]^{\mathrm{cl}}$.

Eq.S3.12 provides the upper bound of selection strength for σ in this model. Noting that $E\left[h(\sigma)\right]^{\mathrm{cl}}$ is always positive,

$$\frac{S\left[\sigma\right]}{S\left[D\right]} = \frac{S\left[\sigma\right]}{2S\left[\sigma\right] + E\left[h(\sigma)\right]^{\mathrm{cl}}}\frac{1}{\ln 2} < \frac{1}{2\ln 2} \approx 0.72 \tag{S3.13}$$

holds. Thus, the selection strength for σ is bounded by $\frac{S[D]}{2\ln 2} \approx 0.72\, S[D]$.

3.2 Selection strength and derivative of long-term population growth rate

Here we briefly discuss the application of large deviation theory [4] to population dynamics and we consider the large deviation property for time-averaged fitness on history. For the convenience of the calculation, we introduce a univariate control parameter β, which perturbs historical fitness. Thereby we define $P_\beta(\sigma)$ as

$$P_\beta(\sigma) \equiv \frac{P^{\mathrm{cl}}(\sigma)e^{\beta H(\sigma)}}{\overline{N}(\tau,\beta)}. \tag{S3.14}$$

where $\overline{N}(\tau,\beta) \equiv \sum_{\sigma\in\mathcal{H}_\tau} P^{\mathrm{cl}}(\sigma)e^{\beta H(\sigma)}$. Evidently, $P_{\beta=0}(\sigma) = P^{\mathrm{cl}}(\sigma)$ and $P_{\beta=1}(\sigma) = P^{\mathrm{rs}}(\sigma)$. We call β historical conditions factor [3]. We assume the existence of long term population growth rate:

$$\Lambda(\beta) \equiv \lim_{\tau\to\infty} \frac{1}{\tau} \ln \overline{N}(\tau,\beta). \tag{S3.15}$$

We call historical fitness divided by time-length τ, $\overline{f}_\tau \equiv H(\sigma)/\tau$, time-averaged fitness ($\overline{f}_\tau$ is a random variable). We define the probability distribution of time-averaged fitness by

$$Q_\beta(h) \equiv \sum_{\sigma\in\mathcal{H}_{\tau,h}} P_\beta(\sigma) \tag{S3.16}$$

where $\mathcal{H}_{\tau,h}$ is a set of histories with length τ and with the time-averaged fitness $\overline{f}_\tau = h$. Thus, chronological and retrospective probabilities are defined by $Q^{\mathrm{cl}}(h) \equiv Q_{\beta=0}(h)$ and $Q^{\mathrm{rs}}(h) \equiv Q_{\beta=1}(h)$. We assume the existence of the limit of time-averaged fitness h^{cl}_* such that

$$\lim_{\tau\to\infty} \overline{f}_\tau = h^{\mathrm{cl}}_* \tag{S3.17}$$

holds with probability 1 in chronological perspective. h^{cl}_* is the most probable time-average fitness with respect to the chronological probability. We further assume the large deviation property of $Q^{\mathrm{cl}}(h)$:

$$Q^{\mathrm{cl}}(h) \approx e^{-\tau I^{\mathrm{cl}}(h)}, \tag{S3.18}$$

for large τ, where $I^{\mathrm{cl}}(h)$ is the rate function that satisfies $I^{\mathrm{cl}}(h) \geq I^{\mathrm{cl}}(h^{\mathrm{cl}}_*) = 0$. $I^{\mathrm{cl}}(h)$ is the convex function of h. Then, the rate function of $Q_\beta(h)$ can be represented as

$$I_\beta(h) \equiv -\lim_{\tau\to\infty} \frac{1}{\tau} \ln Q_\beta(h) = I^{\mathrm{cl}}(h) - \beta h + \Lambda(\beta). \tag{S3.19}$$

Thus, the most probable time-averaged fitness in population with historical condition factor β is

$$h_*(\beta) \equiv \arg\min_h I_\beta(h) = \arg\max_h \left[\beta h - I^{\mathrm{cl}}(h)\right], \tag{S3.20}$$

and the variational formula

$$\Lambda(\beta) = \beta h_*(\beta) - I^{\mathrm{cl}}(h_*(\beta)) = \max_h \left[\beta h - I^{\mathrm{cl}}(h)\right] \tag{S3.21}$$

holds. The stationary condition for the term $\beta h - I^{\mathrm{cl}}(h)$ is

$$\beta = \left.\frac{dI^{\mathrm{cl}}(h)}{dh}\right|_{h=h_*(\beta)}. \tag{S3.22}$$

Evidently, $h_*^{\mathrm{cl}} = h_*(0)$ and we define $h_*^{\mathrm{rs}} \equiv h_*(1)$. Since $I^{\mathrm{cl}}(h)$ is the convex function, by inverse Legendre transformation of Eq.S3.21, we obtain

$$I^{\mathrm{cl}}(h) = \max_\beta \left[\beta h - \Lambda(\beta)\right]. \tag{S3.23}$$

Thus, the rate function of $Q^{\mathrm{rs}}(h)$ is

$$I^{\mathrm{rs}}(h) \equiv I_{\beta=1}(h) = \max_\beta \left[\beta h - \Lambda(\beta)\right] - (h - \Lambda(1)). \tag{S3.24}$$

Using Eq.S3.22, we obtain

$$\frac{d\Lambda(\beta)}{d\beta} = h_*(\beta) + \beta\frac{dh_*(\beta)}{d\beta} - \frac{dh_*(\beta)}{d\beta}\left.\frac{dI^{\mathrm{cl}}(h)}{dh}\right|_{h=h_*(\beta)} = h_*(\beta), \tag{S3.25}$$

and thus

$$h_*^{\mathrm{cl}} = h_*(0) = \left.\frac{d\Lambda(\beta)}{d\beta}\right|_{\beta=0}, \tag{S3.26}$$

and

$$h_*^{\mathrm{rs}} = h_*(1) = \left.\frac{d\Lambda(\beta)}{d\beta}\right|_{\beta=1}. \tag{S3.27}$$

Since $\overline{f}_\tau = H(\sigma)/\tau$ represents the fitness landscape of phenotypic histories, the selection strength for phenotypic histories is represented as

$$S\left[\sigma\right] = \left\langle \overline{f}_\tau \right\rangle^{\mathrm{rs}} - \left\langle \overline{f}_\tau \right\rangle^{\mathrm{cl}} = S\left[\overline{f}_\tau\right]. \tag{S3.28}$$

Thus the large τ limit of $S\left[\sigma\right]$ is exactly calculated as

$$S\left[\overline{f}_\infty\right] \equiv \lim_{\tau\to\infty} S\left[\overline{f}_\tau\right] = h_*^{\mathrm{rs}} - h_*^{\mathrm{cl}} = \left.\frac{d\Lambda(\beta)}{d\beta}\right|_{\beta=1} - \left.\frac{d\Lambda(\beta)}{d\beta}\right|_{\beta=0}. \tag{S3.29}$$

As done in [3], $\Lambda(\beta)$ can be analytically calculated or at least numerically calculated from the results of simulations. Thus, without calculating or sampling specific long-term histories, we can evaluate the long-term limit of the selection strength for phenotypic histories. Such evaluation is done for the following examples.

3.3 Two-state switching model

Here we treat a simplified version of the previous model, in which individuals transits between two phenotypic states, each adapted to two different environments. Adapted individuals have instantaneous fitness f_{a} and non-adapted individuals, lower fitness f_{na}. Individuals can switch phenotypes either stochastically of responsively. Stochastic and responsive switching rates are given by s and s_r, respectively. Adapted individuals switch to the non-adapted state with rate s and non-adapted individuals switch to the adapted state with rate $s + s_r$. Now we consider the situation where the environment changes between two conditions periodically, with the period τ_{env} (i.e. population grows in the same environment during the period τ_{env}).

When q_{a} is the frequency of the adaptive phenotypic state on a history, the fitness landscape for phenotypic history is exactly represented as

$$h\left(q_{\mathrm{a}}\right) = \frac{1}{\tau}\int_0^\tau f\left(x(t)\right) dt = f_{\mathrm{a}} q_{\mathrm{a}} + f_{\mathrm{na}}\left(1 - q_{\mathrm{a}}\right) = f_{\mathrm{na}} + q_{\mathrm{a}}\Delta f, \tag{S3.30}$$

where $\Delta f \equiv f_{\mathrm{a}} - f_{\mathrm{na}}$. In small τ_{env}^{-1} condition where the environment changes as slowly as the population reaches the steady growing state in each environment, we have the analytical solution for $\Lambda(\beta)$ as shown in [3],

$$\Lambda(\beta) = \frac{\beta(f_{\mathrm{a}} + f_{\mathrm{na}}) - (2s + s_r) + K(\beta)}{2} + \frac{1}{\tau_{\mathrm{env}}}\ln\frac{2s + s_r}{K(\beta)} + o(\tau_{\mathrm{env}}^{-1}), \tag{S3.31}$$

where

$$K(\beta) = \sqrt{(\beta\Delta f)^2 + 2(\beta\Delta f)s_r + (2s + s_r)^2}. \tag{S3.32}$$

Higher order terms denoted by $o(\tau_{\mathrm{env}}^{-1})$, which decrease faster that τ_{env}^{-1} as $\tau_{\mathrm{env}} \to \infty$, can be expressed as

$$\frac{1}{\tau_{\mathrm{env}}}\left(c_1 e^{-\tau K(\beta)} + c_2 e^{-2\tau K(\beta)} + \cdots\right), \tag{S3.33}$$

using the powers of $e^{-\tau K(\beta)}$, where $c_1, c_2...$ are constants. Thus, $e^{-\tau K(\beta)} \ll 1$ is the condition with which the expansion of Eq.S3.31 gives a good approximation of $\Lambda(\beta)$. By using the relation

$$\frac{dK(\beta)}{d\beta} = \frac{\Delta f(\beta\Delta f + s_r)}{K(\beta)}, \tag{S3.34}$$

we can derive

$$\frac{d\Lambda(\beta)}{d\beta} = \frac{f_{\mathrm{a}} + f_{\mathrm{na}}}{2} + \Delta f(\beta\Delta f + s_r)\left(\frac{1}{2K(\beta)} - \frac{1}{\tau_{\mathrm{env}}K(\beta)^2}\right) + o(\tau_{\mathrm{env}}^{-1}). \tag{S3.35}$$

Evaluating Eq.S3.35 at $\beta = 0$ and $\beta = 1$, we can obtain the selection strength for phenotypic history in the long-term limit $\tau \to \infty$ from Eq.S3.29. Since the general form of Eq.S3.35 are complicated, we discuss three limiting cases. For each case, only the smallest order term among non-zero terms (both for $O(1)$ and for $O(\tau_{\mathrm{env}}^{-1})$) is remained in the calculations.

For responsive switching where $s_r \gg \Delta f$ and $s/\Delta f \to 0$,

$$h_*^{\mathrm{cl}} = f_{\mathrm{a}} - \frac{1}{\tau_{\mathrm{env}}}\frac{\Delta f}{s_r} + o(\tau_{\mathrm{env}}^{-1}), \tag{S3.36}$$

and

$$S\left[\overline{f}_\infty\right] = \frac{1}{\tau_{\mathrm{env}}}\left(\frac{\Delta f}{s_r}\right)^2 + o(\tau_{\mathrm{env}}^{-1}). \tag{S3.37}$$

In this case, increasing the frequency of environmental changes (i.e. $\tau_{\text{env}} \searrow$) reduces the fitness of the most probable chronological histories while it raises the selection strength. Still, the reduction of chronological fitness is much larger than the fitness gain by the increase of selection strength since $s_r \gg \Delta f$. Thus, increasing the frequency of environmental changes reduces the fitness of the most probable retrospective histories.

For stochastic switching where $s_r = 0$,

$$h_*^{\text{cl}} = \frac{f_{\text{a}} + f_{\text{na}}}{2} + o(\tau_{\text{env}}^{-1}), \tag{S3.38}$$

and thus the change of environmental duration dose not change the fitness of the most probable chronological histories in the order of τ_{env}^{-1}. When $2s \gg \Delta f$ (fast switching),

$$S\left[\overline{f}_\infty\right] = \frac{\Delta f^2}{4s} - \frac{1}{\tau_{\text{env}}}\left(\frac{\Delta f}{2s}\right)^2 + o(\tau_{\text{env}}^{-1}). \tag{S3.39}$$

Therefore, the increased frequency of environmental changes reduces the selection strength. When $2s \ll \Delta f$ (slow switching),

$$S\left[\overline{f}_\infty\right] = \frac{\Delta f}{2} - \frac{1}{\tau_{\text{env}}} + o(\tau_{\text{env}}^{-1}), \tag{S3.40}$$

and again, the selection strength decreases.

This two-state switching model was already studied in [3], in which another measure of selection strength, $\mathcal{M}_s$, was introduced. $\mathcal{M}_s$ is defined as

$$\mathcal{M}_s \equiv \lim_{\tau\to\infty} \tau \text{Var}\left[\overline{f}_\tau\right]^{\text{rs}}. \tag{S3.41}$$

This is also expressed as

$$\mathcal{M}_s = \left.\frac{d^2\Lambda(\beta)}{d\beta^2}\right|_{\beta=1}. \tag{S3.42}$$

Thus, we can directly compare $S\left[\overline{f}_\infty\right]$ with $\mathcal{M}_s$ by calculating the second derivative of $\Lambda(\beta)$ and evaluating it at $\beta = 1$.

In the cases of weak selection (the first and second cases), we can show that the representations becomes identical between $S\left[\overline{f}_\infty\right]$ and $\mathcal{M}_s$ to first order in τ_{env}^{-1}. In the third case ($s_r = 0, 2s \ll \Delta f$), the representations are slightly different:

$$\mathcal{M}_s = \frac{2s^2}{\Delta f} + \frac{1}{\tau_{\text{env}}} + o(\tau_{\text{env}}^{-1}) \tag{S3.43}$$

(see Eq.S3.40). The difference between $S\left[\overline{f}_\infty\right]$ and $\mathcal{M}_s$ means that the second derivative of $\Lambda(\beta)$ greatly changes for $0 \leq \beta \leq 1$ because we can express $S\left[\overline{f}_\infty\right]$ as

$$S\left[\overline{f}_\infty\right] = \int_0^1 \frac{d^2\Lambda(\beta)}{d\beta^2} d\beta. \tag{S3.44}$$

In fact, when $s_r = 0$,

$$\frac{d^2\Lambda(\beta)}{d\beta^2} = \frac{1}{2}\frac{\Delta f^2}{\sqrt{(\beta\Delta f)^2 + (2s)^2}}\frac{(2s)^2}{(\beta\Delta f)^2 + (2s)^2} + \frac{1}{\tau_{\text{env}}}\frac{\Delta f^2}{(\beta\Delta f)^2 + (2s)^2}\frac{(\beta\Delta f)^2 - (2s)^2}{(\beta\Delta f)^2 + (2s)^2}. \tag{S3.45}$$

This is more sensitive to the changes in β when $2s \ll \Delta f$ than when $2s \gg \Delta f$.

3.4 Simple stochastic gene expression model

In this last section, we apply our framework to an analytically tractable stochastic gene expression model.

Here we treat the continuous-state stochastic process. We assume that protein is produced from mRNA at a constant rate k_p, and mRNA from gene at a rate k [5, 6]. We also assume that life time (due to dilution + degradation) of mRNA, γ_m^{-1}, is much shorter than that of protein, γ^{-1}. In the limit of $\gamma/\gamma_m \to 0$ with $b = k_p/\gamma_m$ fixed, protein copy number produced per mRNA transcript follows exponential distribution with mean b (called mean burst size). Let X_t be protein copy number at time t. Then,

$$E\left[dX_t | X_t = x\right] = (kb - \gamma x)dt, \tag{S3.46}$$

and

$$E\left[dX_t^2 | X_t = x\right] = 2kb^2 dt. \tag{S3.47}$$

We introduce diffusion approximation (i.e. $E[dX_t^n] = 0$ for $n \geq 3$) [7, 8] (master equation without this approximation is described in [9]; its stationary solution is gamma distribution). The stochastic differential equation is

$$dX_t = (kb - \gamma X_t)dt + \sqrt{2kb^2}dW_t, \tag{S3.48}$$

where dW_t is the standard Wiener process, and the master equation (Fokker-Planck equation) is,

$$\frac{\partial q(x,t)}{\partial t} = -\frac{\partial}{\partial x}\left[(kb - \gamma x)q(x,t) - kb^2 \frac{\partial}{\partial x} q(x,t)\right], \tag{S3.49}$$

where $q(x,t) = E\left[\delta(X_t - x)\right]$. The stationary solution $q_{\mathrm{st}}(x)$ is

$$q_{\mathrm{st}}(x) = \frac{1}{\sqrt{2\pi \frac{kb^2}{\gamma}}} \exp\left[-\frac{(x - \frac{kb}{\gamma})^2}{2\frac{kb^2}{\gamma}}\right]. \tag{S3.50}$$

This is the stationary protein copy number distribution when difference of copy number causes no fitness advantage or disadvantage for cells (i.e. without selection).

When fitness is correlated with protein copy number, the stationary distribution becomes different from $q_{\mathrm{st}}(x)$. As a simple case, we now assume that instantaneous reproduction rate is a linear function of X_t, i.e.

$$f(X_t) = f_0 + \xi X_t. \tag{S3.51}$$

$\xi = 0$ corresponds to the case without selection. Let $p(x,t) = E\left[\delta(X_t - x)\right]_{\xi}$. The master equation is

$$\frac{\partial p(x,t)}{\partial t} = -\frac{\partial}{\partial x}\left[(kb - \gamma x)p(x,t) - kb^2 \frac{\partial}{\partial x} p(x,t)\right] + \left(f(x) - \lambda(t)\right)p(x,t), \tag{S3.52}$$

where $\lambda(t) \equiv \int f(x)p(x,t)dx$ is instantaneous population fitness at time t [7, 8]. At stationary state, the master equation becomes

$$\lambda_{\mathrm{st}}(\xi)p_{\mathrm{st}}(x) = f(x)p_{\mathrm{st}}(x) - \frac{\partial}{\partial x}\left[(kb - \gamma x)p_{\mathrm{st}}(x) - kb^2 \frac{\partial}{\partial x} p_{\mathrm{st}}(x)\right], \tag{S3.53}$$

where $\lambda_{\rm st}(\xi) = \int f(x)p_{\rm st}(x)dx$. Then, the solution is

$$p_{\rm st}(x) = \frac{1}{\sqrt{2\pi\frac{kb^2}{\gamma}}}\exp\left[-\frac{(x-\mu(\xi))^2}{2\frac{kb^2}{\gamma}}\right], \tag{S3.54}$$

where

$$\mu(\xi) = \frac{kb}{\gamma} + \frac{kb^2}{\gamma^2}\xi, \tag{S3.55}$$

and

$$\lambda_{\rm st}(\xi) = f_0 + \frac{kb}{\gamma}\xi + \frac{kb^2}{\gamma^2}\xi^2 \tag{S3.56}$$

[7, 8].

Now we evaluate the selection strength $S\left[\overline{X}_\infty\right]$. Since instantaneous fitness is a deterministic function of X_t in this model, Eq.S3.29 can be used to obtain $S\left[\overline{X}_\infty\right]$. $\Lambda(\beta)$ defined in Eq.S3.15 is obtained by transforming $f(x) \to \beta f(x)$ in the present model. Replacing f_0 with βf_0 and ξ with $\beta\xi$ in Eq.S3.56, we obtain

$$\Lambda(\beta) = \beta f_0 + \frac{kb}{\gamma}\beta\xi + \frac{kb^2}{\gamma^2}\beta^2\xi^2 \tag{S3.57}$$

Thus,

$$h_*^{\rm cl} = \left.\frac{d\Lambda(\beta)}{d\beta}\right|_{\beta=0} = f_0 + k\rho, \tag{S3.58}$$

$$h_*^{\rm rs} = \left.\frac{d\Lambda(\beta)}{d\beta}\right|_{\beta=1} = f_0 + k\rho + 2k\rho^2, \tag{S3.59}$$

and

$$S\left[\overline{X}_\infty\right] = S\left[\overline{f}_\infty\right] = h_*^{\rm rs} - h_*^{\rm cl} = 2k\rho^2, \tag{S3.60}$$

where

$$\rho = \frac{b\xi}{\gamma}. \tag{S3.61}$$

is the ratio of the timescale of phenotypic fluctuation, γ^{-1}, to the timescale of selection, $(b\xi)^{-1}$. Based on these results, we can tell to what extents an environmental perturbation provokes individual response and change in selection strength. For example, when an environmental perturbation changes only transcription rate k, the individual response and the change in selection strength are

$$\frac{\partial h_*^{\rm cl}}{\partial k} = \rho, \tag{S3.62}$$

and

$$\frac{\partial}{\partial k}S\left[\overline{X}_\infty\right] = 2\rho^2, \tag{S3.63}$$

respectively. Therefore, when $\rho > 1/2$, the change in selection strength contributes to the change in $h_*^{\rm rs}$ to a greater extent than the individual response. Note that we cannot distinguish these two effects only from the changes in $h_*^{\rm rs}$ nor in $\Lambda(\beta)$.

We can also calculate the maximum selection strength $S[D]$ in this model. From. Eq.S3.12, S3.58, and S3.60,

$$S[D] = \left(4k\rho^2 + k\rho + f_0\right) \ln 2. \quad \text{(S3.64)}$$

Thus, the effect of perturbation on transcription rate k on $S[D]$ is

$$\frac{\partial}{\partial k} S[D] = \left(4\rho^2 + \rho\right) \ln 2. \quad \text{(S3.65)}$$

4 Materials and Methods

4.1 Plasmids

We constructed pTN001 and pTN002 plasmids from pLVK3 plasmid in the lab collection. pLVK3 plasmid was constructed by introducing $P_{\text{LlacO-1}}$ promoter [10], *venus* structural gene [11], t1t2 *rrnB* terminator, and frt-franked kanamycin resistance (*kan*^{R}) cassette [12] into the multi-cloning site of pMW118 (pSC101 ori, Nippon Gene).

pLVK3 plasmid harbors SacI-MluI restriction sites within the structural gene of *venus* before the stop codon. We used these sites to introduce streptomycin resistance gene *sm*^{R} into the plasmid pLVK3. We amplified *sm*^{R} gene from pKP2375 plasmid (provided from Hironori Niki, National Institute of Genetics, Japan) by PCR with the primers SacI-str_F and str-Mlu_R (see S3 Table for the primer sequence). The PCR product and pLVK3 were digested by SacI and MluI restriction enzymes, and ligated to obtain pLVSK3.

We also replaced ribosome-binding site (RBS, AGGAGAAAGGTACC) for *venus-sm*^{R} into a stronger one (AGGAGGAAAAAA) by PCR with the primers RBS-A-venus_F and PLseries_R. The PCR product was self-ligated to obtain pLVSK4.

To create pTN001 plasmid, we separated the *venus* and *sm*^{R} structural genes by adding the stop codon to *venus* and placing the RBS (the same sequence as that for *venus*) before *sm*^{R}. We accomplished this by PCR using pLVSK4 as the template and with the primers RBS4ATG_F and TAAA_R. The PCR product was self-ligated to obtain pTN001.

We constructed the control plasmid pTN002 as follows. We first changed the RBS of pLVK3 by PCR with the primers RBS-A-venus_F and PLseries_R. The PCR product was self-ligated to obtain pLVK4 plasmid. Next, we removed the SacI-MluI sites before the stop codon of venus by PCR with the primers term_F and venus_R. The PCR product was again self-ligated to obtain pTN002.

The DNA sequences of pTN001, pTN002, pLVK3, pLVSK4, and pTmCherry4 (see below) are available on the Dryad data repository site.

4.2 E. coli strains

We transformed *E. coli* F3 strain (W3110 Δ*fliC* Δ*fimA* Δ*flu*) [13] with pTN001 and pTN002 to construct F3/pTN001 and F3/pTN002.

We also constructed F3NW strain (W3110 Δ*fliC* Δ*fimA* Δ*flu* *intC*::$P_{LlacO-1}$-*venus-sm*^{R} *galK*::$P_{LtetO-1}$-*mcherry*) using the standard λ-Red recombination method [12]. We first amplified the DNA fragment from P_LlacO-1 promoter to frt-franked *kan*^{R} cassette on pLVSK4 by PCR with the primers intC_PlacO1_F3 and intC_R. The PCR product was introduced into the *intC* locus of *E. coli* BW25113/pKD46 strain (provided from The Coli Genetic Stock Center (CGSC) at Yale) by electroporation. The inserted DNA fragment was transferred to F3 by P1 transduction. *kan*^{R} gene was removed by pCP20 (provided from CGSC at Yale).

We also introduced $P_{\text{LtetO-1}}$-*mcherry* fragment into the *galK* locus, first in BW25113/pKD46, and then transferred to F3 *intC*::$P_{LlacO-1}$-*venus*-sm^{R} by P1 transduction. The DNA fragment of $P_{\text{LtetO-1}}$ [10], *mcherry*, and frt-franked kan^{R} cassette was amplified from pTmCherryK4 plasmid in the lab collection with the primers galK_PtetO1_F3 and galK_R3. The kan^{R} gene was removed by pCP20 to obtain the final construct F3NW.

Supporting References

[1] Bowsher CG, Swain PS. Identifying sources of variation and the flow of information in biochemical networks. Proc Natl Acad Sci U S A. 2012;109(20):E1320–8.

[2] Hermisson J, Redner O, Wagner H, Baake E. Mutation-selection balance: ancestry, load, and maximum principle. Theor Popul Biol. 2002;62(1):9–46.

[3] Leibler S, Kussell E. Individual histories and selection in heterogeneous populations. Proc Natl Acad Sci U S A. 2010;107(29):13183–8.

[4] Dembo A, Zeitouni O. Large Deviations Techniques and Applications. vol. 38 of Stochastic Modelling and Applied Probability. 2nd ed. Berlin, Heidelberg: Springer; 1998.

[5] Shahrezaei V, Swain PS. Analytical distributions for stochastic gene expression. Proc Natl Acad Sci U S A. 2008;105(45):17256–61.

[6] Thattai M, van Oudenaarden a. Intrinsic noise in gene regulatory networks. Proc Natl Acad Sci U S A. 2001;98(15):8614–9.

[7] Sato K, Kaneko K. On the distribution of state values of reproducing cells. Phys Biol. 2006;3(1):74–82.

[8] Mora T, Walczak AM. Effect of phenotypic selection on stochastic gene expression. J Phys Chem B. 2013;117(42):13194–205.

[9] Friedman N, Cai L, Xie XS. Linking Stochastic Dynamics to Population Distribution: An Analytical Framework of Gene Expression. Phys Rev Lett. 2006;97(16):1–4.

[10] Lutz R, Bujard H. Independent and tight regulation of transcriptional units in Escherichia coli via the LacR / O , the TetR / O and AraC / I 1 -I 2 regulatory elements. Nucleic Acids Res. 1997;25(6):1203–1210.

[11] Nagai T, Ibata K, Park ES, Kubota M, Mikoshiba K, Miyawaki A. A variant of yellow fluorescent protein with fast and efficient maturation for cell-biological applications. Nat Biotechnol. 2002;20(1):87–90.

[12] Datsenko Ka, Wanner BL. One-step inactivation of chromosomal genes in Escherichia coli K-12 using PCR products. Proc Natl Acad Sci U S A. 2000;97(12):6640–5.

[13] Hashimoto M, Nozoe T, Nakaoka H, Okura R, Akiyoshi S, Kaneko K, et al. Noise-driven growth rate gain in clonal cellular populations. Proc Natl Acad Sci. 2016;113(12):3251–3256.
