# Supplementary material for: Inferring fitness landscapes and selection on phenotypic states from single-cell genealogical data
Source: PLoS Genet. 2017 Mar 7;13(3):e1006653. doi: 10.1371/journal.pgen.1006653 (PMC5360348; doi:10.1371/journal.pgen.1006653)
Supplement: S1 Table — (PDF) [file pgen.1006653.s002.pdf]

Data	0 min	200 min	400 min
− Sm #1	235	1042	4752
− Sm #2	148	721	3298
− Sm #3	89	394	1622
+ Sm #1	190	804	2828
+ Sm #2	172	793	2995
+ Sm #3	137	593	2239
