# Supplementary material for: Inferring fitness landscapes and selection on phenotypic states from single-cell genealogical data
Source: PLoS Genet. 2017 Mar 7;13(3):e1006653. doi: 10.1371/journal.pgen.1006653 (PMC5360348; doi:10.1371/journal.pgen.1006653)
Supplement: S2 Table — (PDF) [file pgen.1006653.s003.pdf]

Data	0 min	100 min	300 min
− Sm #1	70	171	952
− Sm #2	77	192	1082
− Sm #3	54	121	644
− Sm #4	110	260	1694
+ Sm #1	147	298	1486
+ Sm #2	55	137	645
+ Sm #3	42	92	477
+ Sm #4	58	125	652
