# Supplementary material for: Inferring fitness landscapes and selection on phenotypic states from single-cell genealogical data
Source: PLoS Genet. 2017 Mar 7;13(3):e1006653. doi: 10.1371/journal.pgen.1006653 (PMC5360348; doi:10.1371/journal.pgen.1006653)
Supplement: S3 Table — (PDF) [file pgen.1006653.s004.pdf]

Name	Sequence (5' to 3')
SacI-str_F	AAAGAGCTCCGCTCACGCAACTGGTCCAGAAC
str-Mlu_R	CCAACGCGTTTTGCCGACTACCTTGGTGATC
RBS-A-venus_F	GGAAAAAAATGGTGAGCAAGGGCGAGGAG
PLseries_R	TCCTCTTTAATGTTTTCGGTCAGTGC
RBS4ATG_F	AGGAGGAAAAAAATGCGCTCACGCAACTGGTCCAGAA
TAAA_R	TTTACTTGTACAGCTCGTCCATGCCG
term_F	CAGGCATCAAATAAAACGAAAGGC
venus_R	TTACTTGTACAGCTCGTCCAT
intC_PlacO1_F3	AGTTGTTAAGGTCGCTCACTCCACCTTCTCATCAAGCC AGTCCGCCCAAATTGTGAGCGGATAACAATTG
intC_R	CCGTAGATTTACAGTTCGTCATGGTTCGCTTCAGATCG TTGACAGCCGCAATTCCGGGGATCCGTCGACC
galK_PtetO1_F3	TTCATATTGTTCAGCGACAGCTTGCTGTACGGCAGGCA CCAGCTCTTCCGAATTGTGAGCGGATAACAAT
galK_R3	GTTTGCGCGCAGTCAGCGATATCCATTTTCGCGAATCC GGAGTGTAAGAAATTCCGGGGATCCGTCGACC
