# Supplementary figures and images for: Inferring fitness landscapes and selection on phenotypic states from single-cell genealogical data
Source: PLoS Genet. 2017 Mar 7;13(3):e1006653. doi: 10.1371/journal.pgen.1006653 (PMC5360348; doi:10.1371/journal.pgen.1006653)

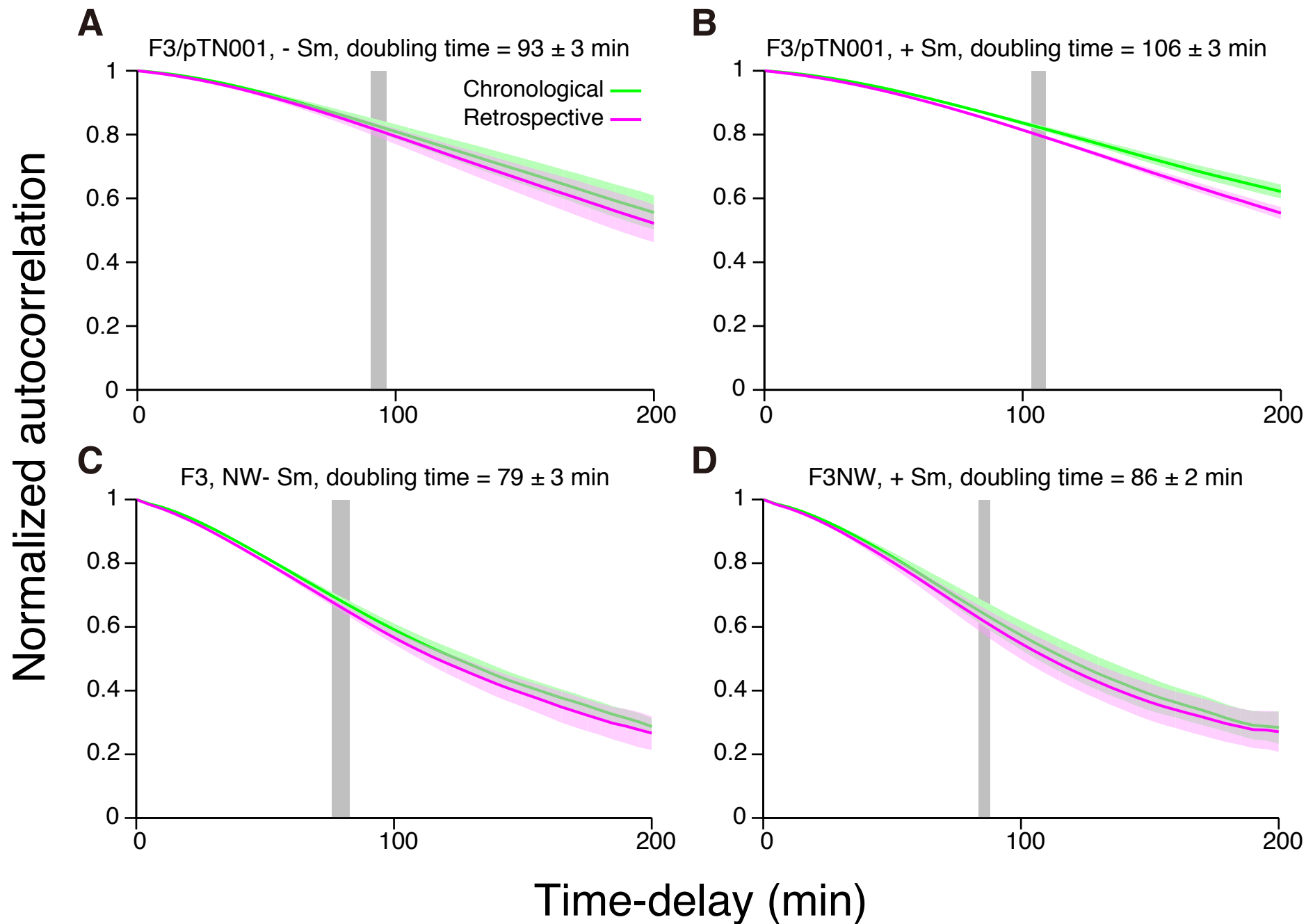

Supplement: S1 Fig — Normalized autocorrelation functions of experimental data were calculated according to the method described in section 2.8 in S1 Text. Autocorrelation functions with chronological weights are shown by green solid lines and those with retrospective weights are shown by magenta solid lines. Colored shades for each line indicate the ± standard deviation over all the independent measurements for each pair of strain and drug condition (3 for A and B, and 4 for C and D). The gray time windows indicate the doubling time with ± standard deviation over all the independent measurements. The doubling time was calculated by dividing ln2 by the population growth rate in Eq.S2.11 in S1 Text. A. F3/pTN001 without streptomycin, B. F3/pTN001 with 200 μg/mL streptomycin, C. F3NW without streptomycin, D. F3NW with 100 μg/mL streptomycin. These graphs show that the autocorrelation decays to approximately 0.8 in one generation for F3/pTN001 and 0.7 for F3NW. (PDF) [file pgen.1006653.s005.pdf]

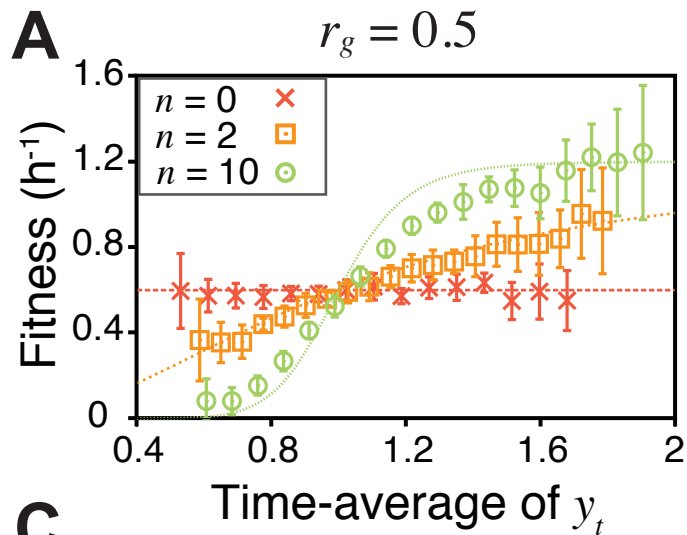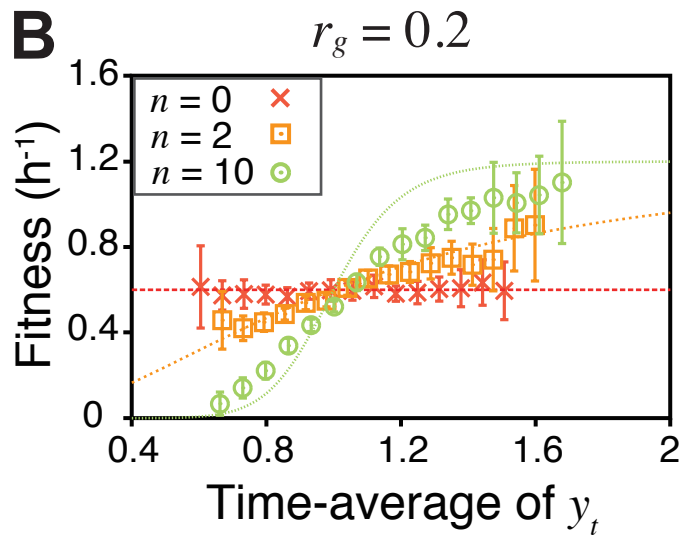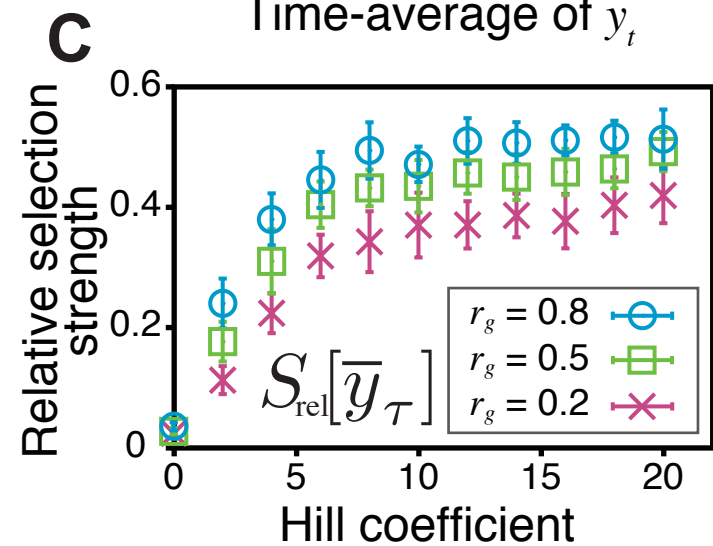

Supplement: S2 Fig — A. Fitness landscapes for rg = 0.5. We produced the datasets of clonal cell proliferation by simulation, in which we assumed that cells stochastically change phenotypic state y and divide in a phenotype-dependent manner with the division rate f(y)=yn1+ynfmax (broken curves). We calculated fitness landscapes h(y¯) from the simulation data for the conditions of n = 0, 2, and 10. The points and the error bars represent the means and the standard deviations of the results from 10 independent simulations. B. Fitness landscapes for rg = 0.2. C. Dependence of relative selection strength Srel[y¯τ]=S[y¯τ]/S[D] on hill coefficient for rg = 0.2, 0.5 and 0.8. As rg decreases, the relative selection strength also decreased for the same value of hill coefficient. (PDF) [file pgen.1006653.s006.pdf]

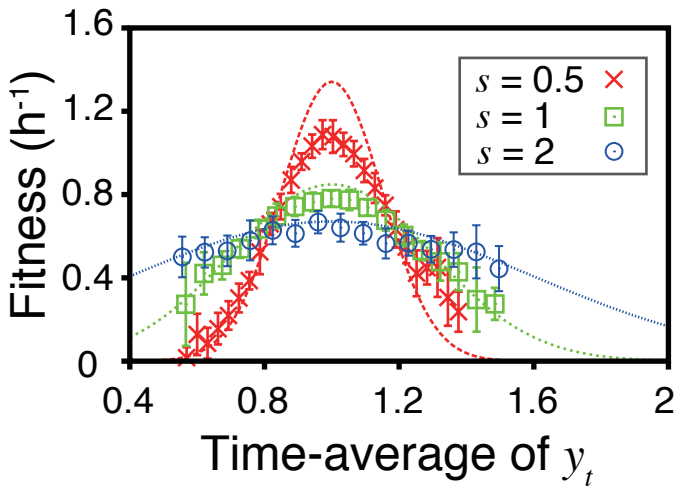

Supplement: S3 Fig — Fitness landscapes for rg = 0.8 with Gaussian fitness functions. We produced the datasets of clonal cell proliferation by simulation, in which we assumed that cells stochastically change phenotypic state y and divide in a phenotype-dependent manner with the division rate f(y)=f01+s-2exp(-(y-1)2/2(0.3s)2) (broken curves). We calculated fitness landscapes h(y¯) from the simulation data for the conditions of s = 0.5, 1, and 2. The points and the error bars represent the means and the standard deviations of the results from 10 independent simulations. (PDF) [file pgen.1006653.s007.pdf]

**A**

|            | − SM  |       |       |                  | + SM  |       |       |                  |
|------------|-------|-------|-------|------------------|-------|-------|-------|------------------|
| Time (min) | #1    | #2    | #3    | Ave.±SD          | #1    | #2    | #3    | Ave.±SD          |
| 200        | 0.06  | 0.01  | 0.13  | $0.06 \pm 0.05$  | 0.06  | 0.15  | 0.06  | $0.09 \pm 0.04$  |
| 300        | 0.00  | −0.05 | 0.05  | $0.00 \pm 0.04$  | −0.03 | −0.05 | −0.08 | $−0.05 \pm 0.02$ |
| 400        | −0.07 | −0.11 | −0.03 | $−0.07 \pm 0.03$ | −0.15 | −0.11 | −0.18 | $−0.14 \pm 0.03$ |

**B**

−SM #2, t = 300 min, N = 1567

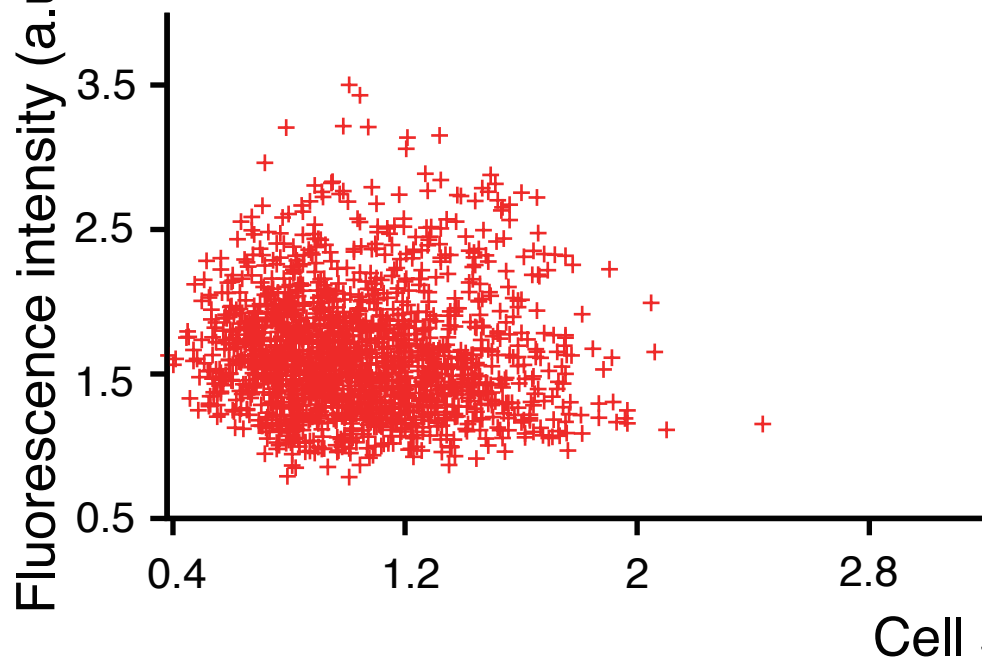**C**

+SM #2, t = 300 min, N = 1610

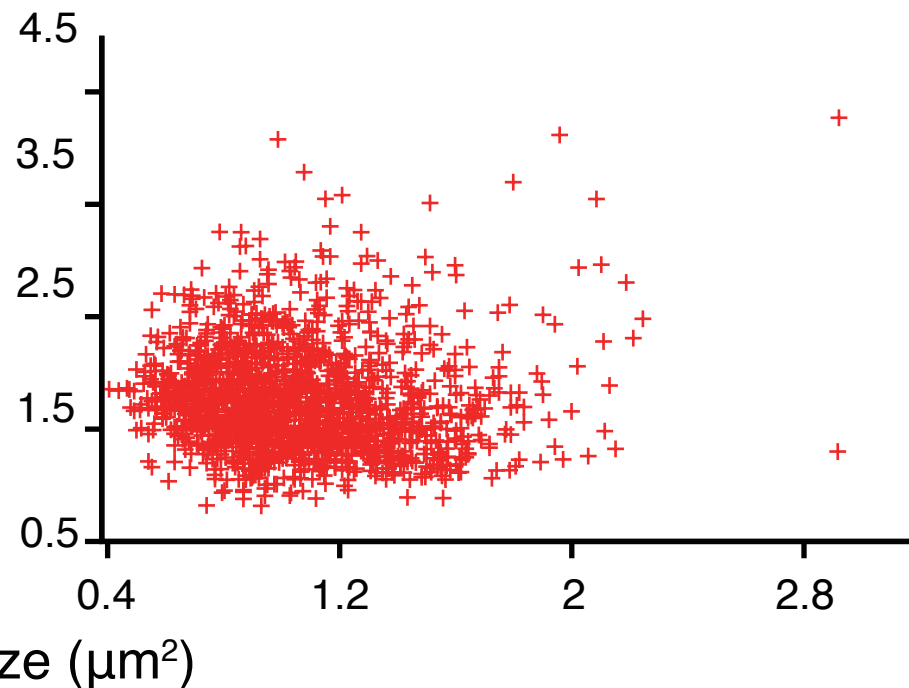

Supplement: S4 Fig — A. Correlation coefficients between fluorescence intensity c and cell size v in the data of F3/pTN001 for −Sm and +Sm conditions at three different time points, 200, 300 and 400 min. Averages and standard deviations of the correlation coefficients among three independent measurements for each drug condition are also shown. Those data suggest that c and v are almost uncorrelated. Typical scatter plots of c vs v are shown in B and C. B. Scatter plot of c vs v for the measurement #2 with −Sm condition for F3/pTN001 at 300 min. C. Scatter plot of c vs v for the measurement #2 with +Sm condition for F3/pTN001 at 300 min. (PDF) [file pgen.1006653.s008.pdf]

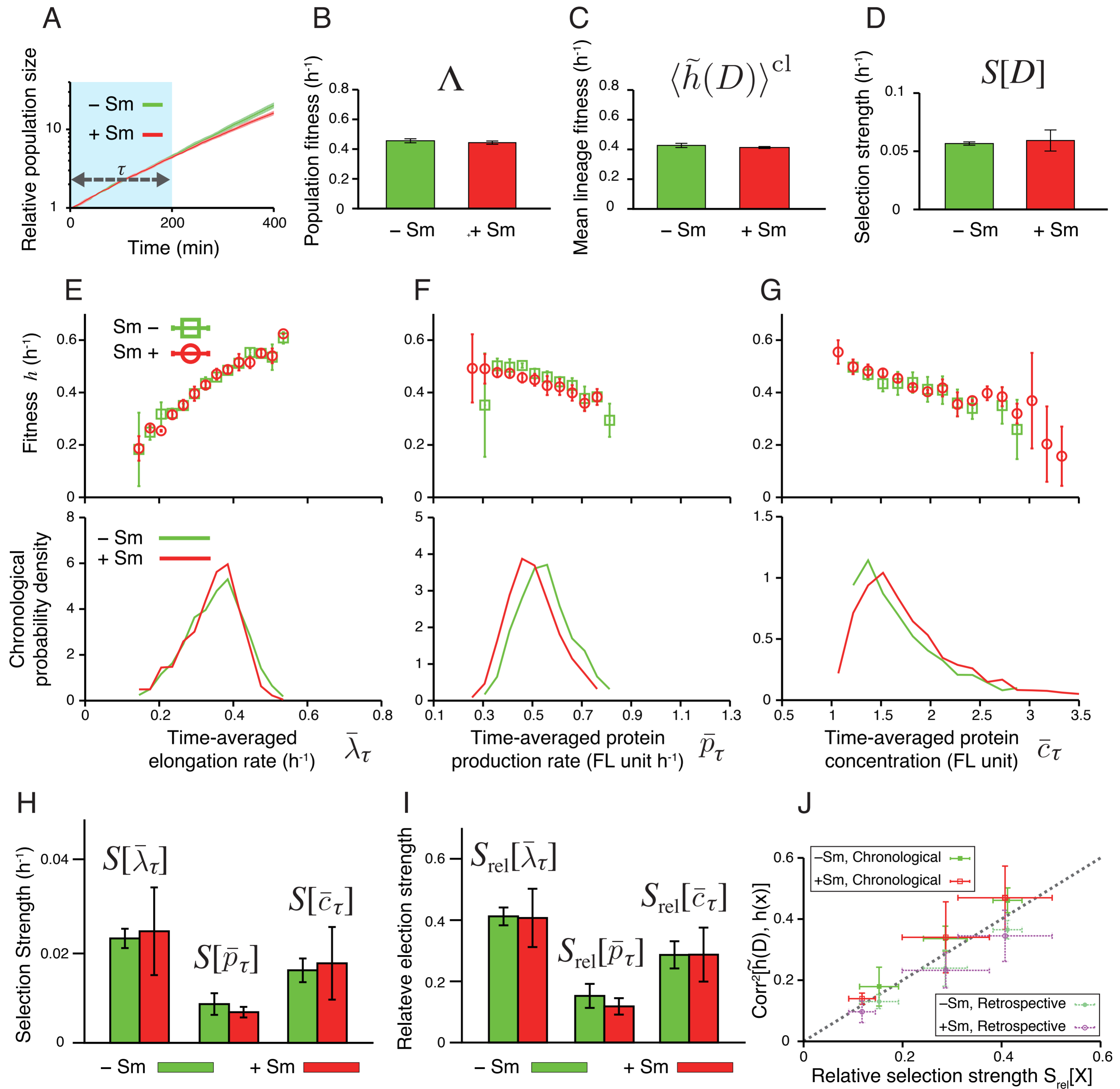

Supplement: S6 Fig — A. Population growth curves. The time window colored in light blue corresponds to the early term. Green curve is for −Sm condition, and red for +Sm condition (the color correspondence is the same for all the following panels). Relative population size on Y-axis is the number of cells at each time point normalized by the number of cells at t = 0 min. The error bars are the standard deviations of three independent experiments, which is also true for all the error bars in the following panels. B. Comparison of population growth rate between +Sm and −Sm conditions. Error bars are SD of the three replicate experiments (the same for all the results below). C. Comparison of the mean fitness 〈h(D)〉cl. D. Comparison of selection strength S[D]. E-G. Fitness landscapes and chronological probability distributions of the phenotypes: elongation rate (E), protein production rate (F), and protein concentration (G). H. Comparison of selection strength between +Sm and −Sm conditions. I. Relative selection strengths. J. Relationship between relative selection strength and squared correlation coefficient between h˜(D) and h(x), where x=λ¯τ, p¯τ, or c¯τ. The correlation coefficients were evaluated by both chronological and retrospective probabilities. (PDF) [file pgen.1006653.s010.pdf]

# F3/pTN001 (200 - 400 min)

– Sm

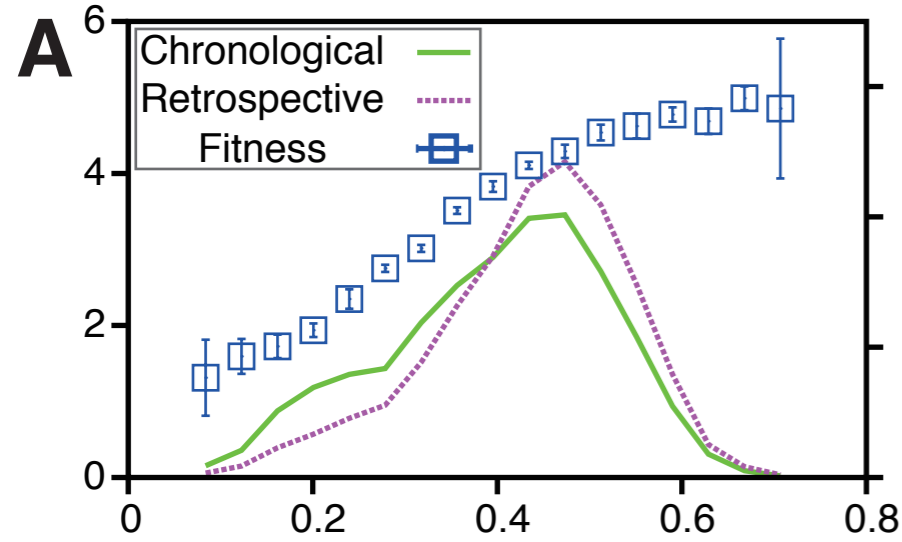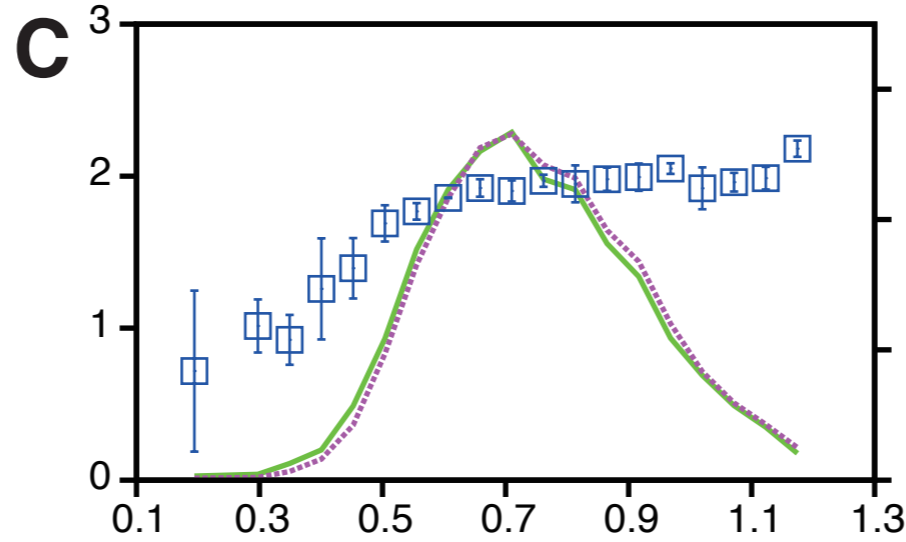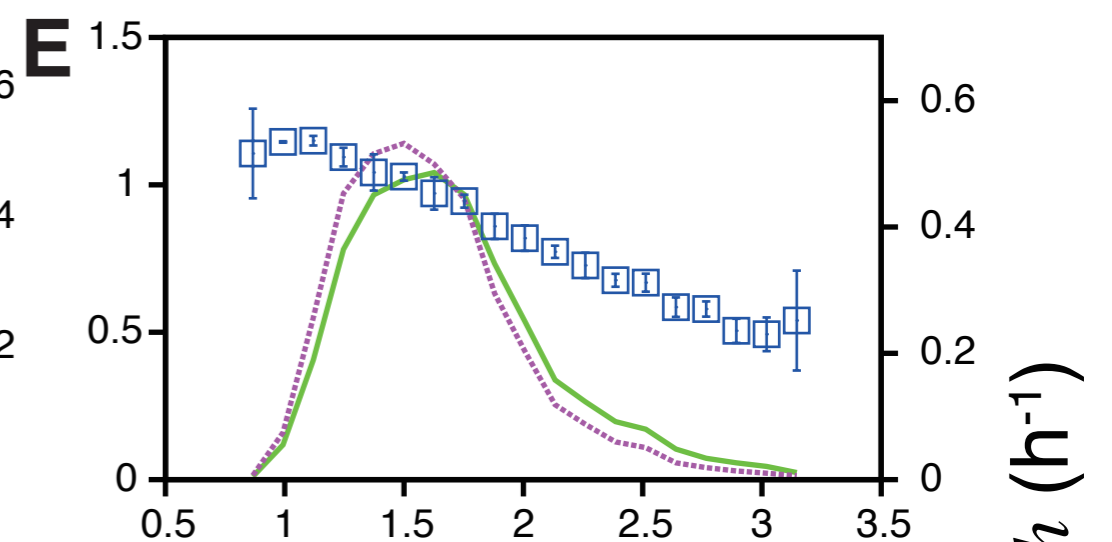

+ Sm

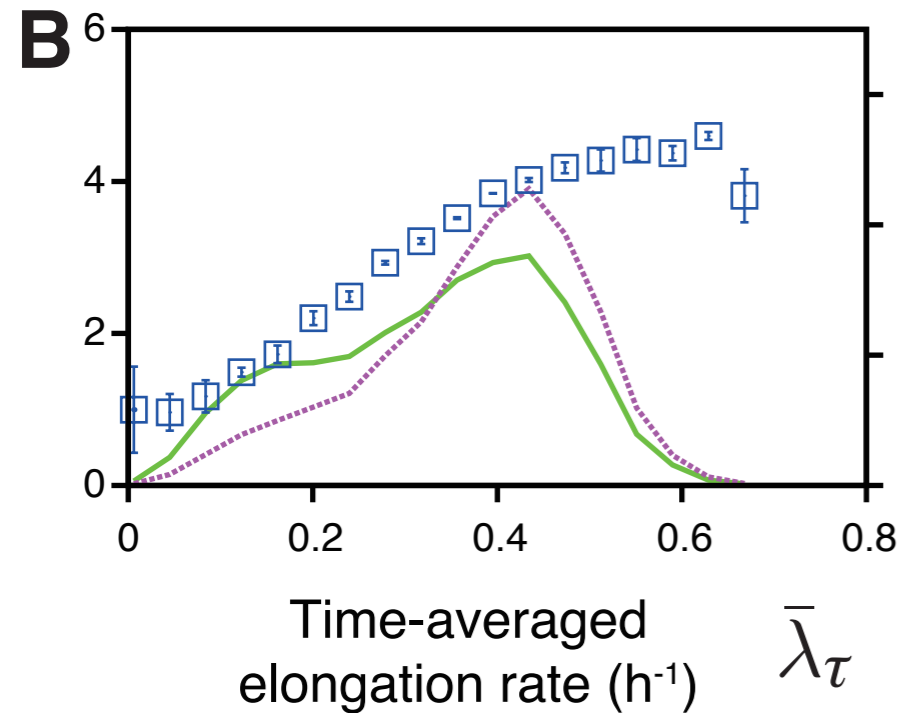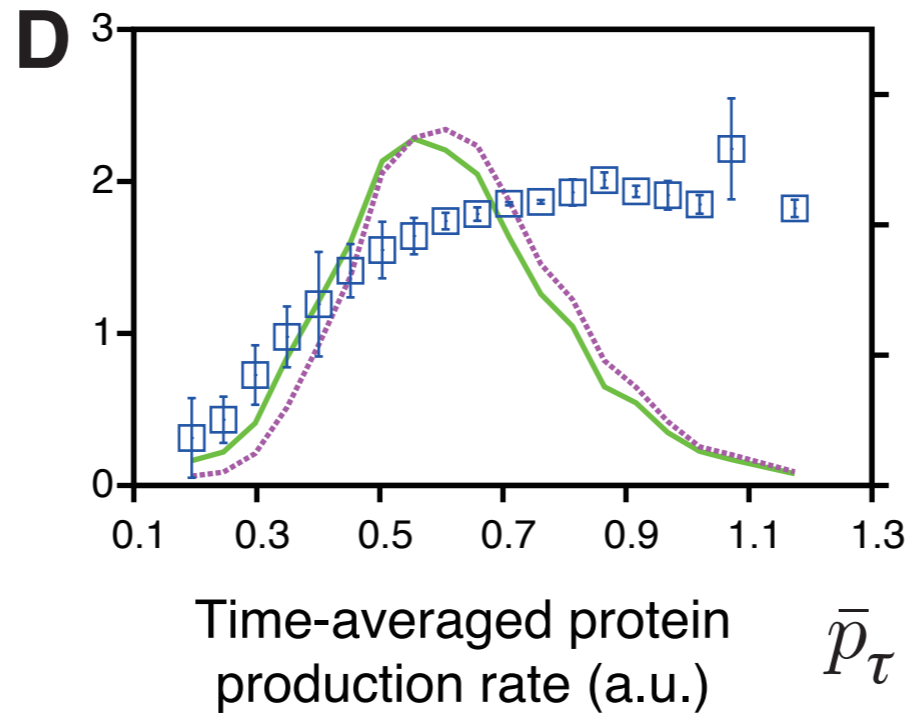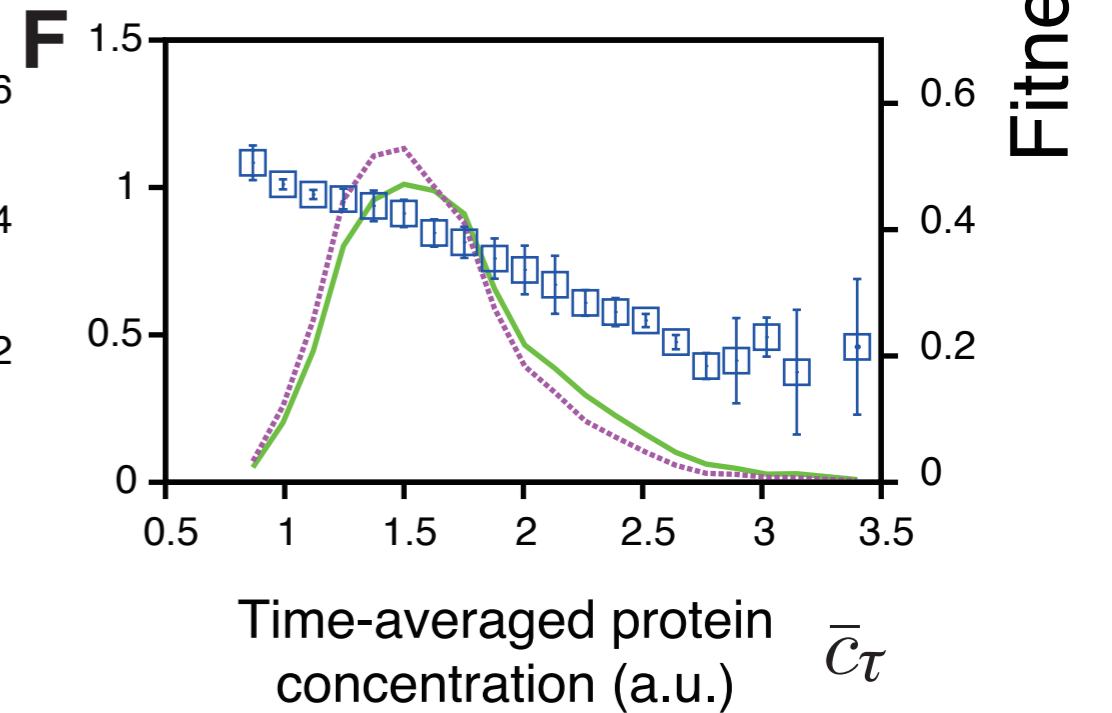

Supplement: S7 Fig — In each panel, chronological probability distribution (green), retrospective probability distribution (magenta), and fitness landscape (blue) are shown. The error bars for the fitness landscapes represent ± standard deviations among the replicate experiments. A. Time-averaged elongation rate, −Sm. B. Time-averaged elongation rate, +Sm. C. Time-averaged protein production rate, −Sm. D. Time-averaged protein production rate, +Sm. E. Time-averaged protein concentration, −Sm. F. Time-averaged protein concentration, +Sm. (PDF) [file pgen.1006653.s011.pdf]

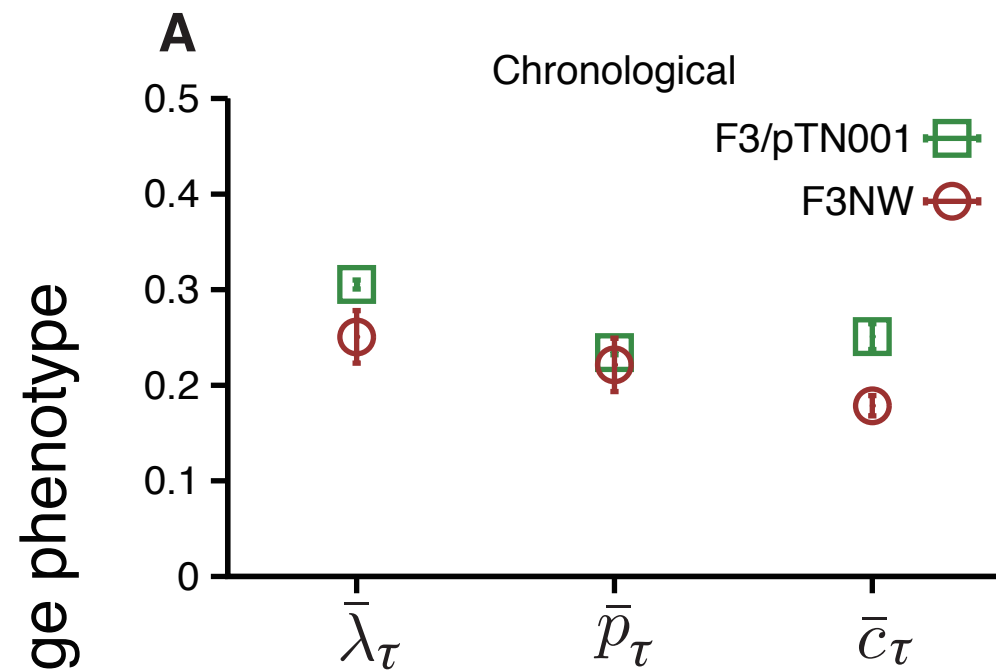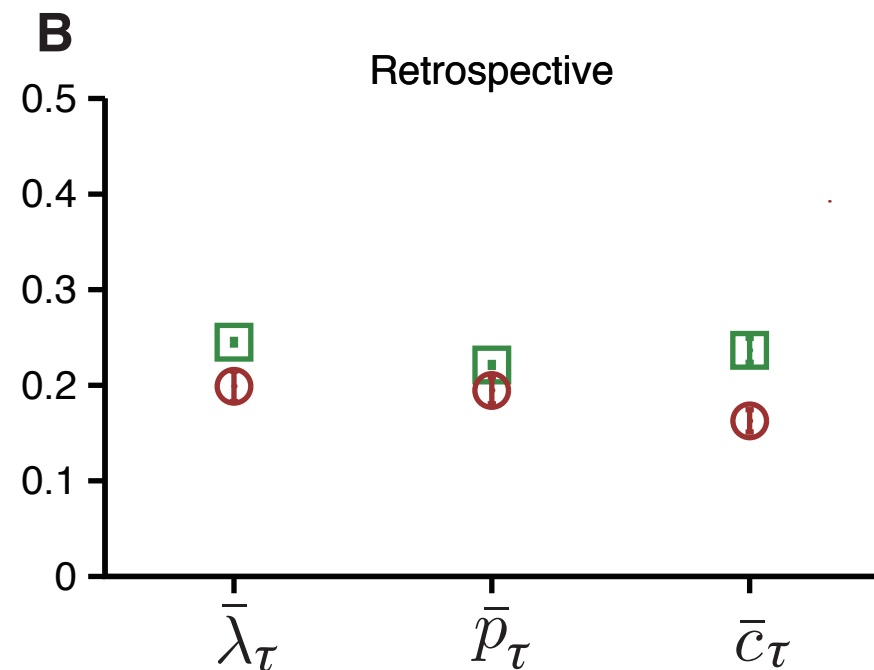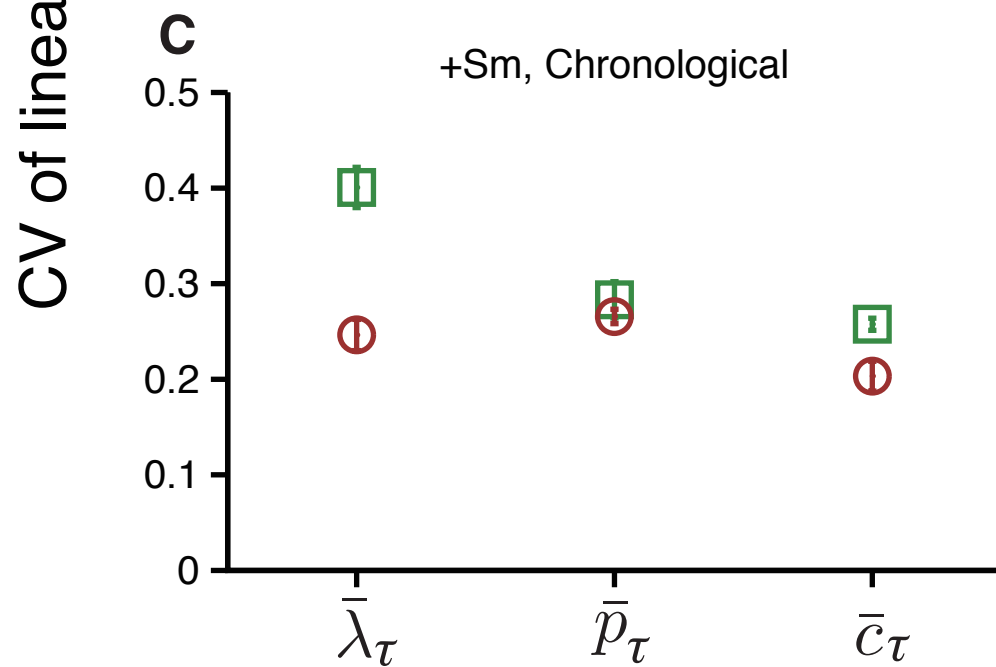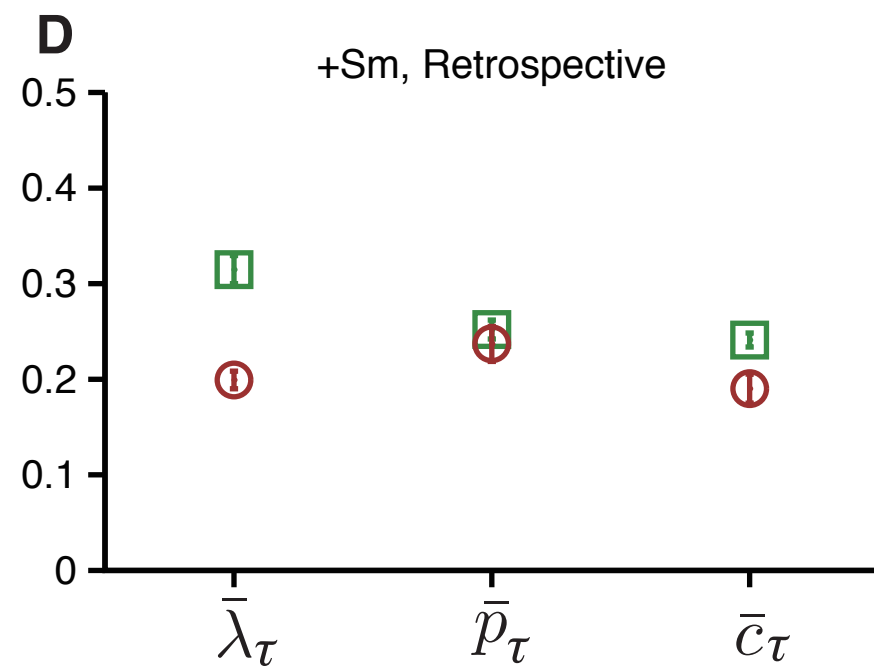

Supplement: S9 Fig — The CVs for time-averaged elongation rate λ¯τ, time-averaged protein production rate p¯τ and time-averaged protein concentration c¯τ are calculated with both chronological and retrospective weighing. The CVs for F3/pTN001 (green squares) and those for F3NW (brown circles) are compared in each panel: (A) chronological, −Sm, (B) retrospective, −Sm, (C) chronological, +Sm, (D) retrospective, +Sm. Error bars are the standard deviations among 3 or 4 independent measurements (3 for F3/pTN001 and 4 for F3NW). The CVs for F3NW were not greater than those for F3/pTN001 in all the cases. (PDF) [file pgen.1006653.s013.pdf]

Selection strength ( $h^{-1}$ )

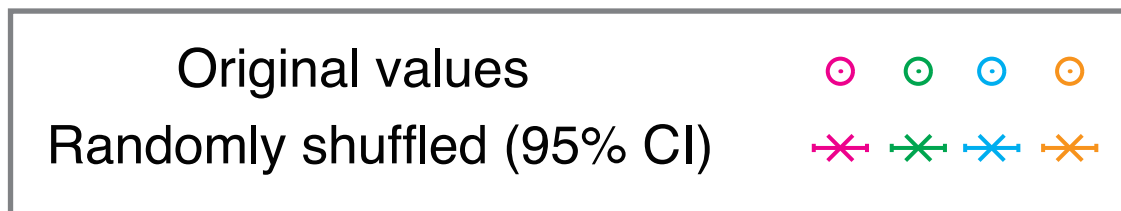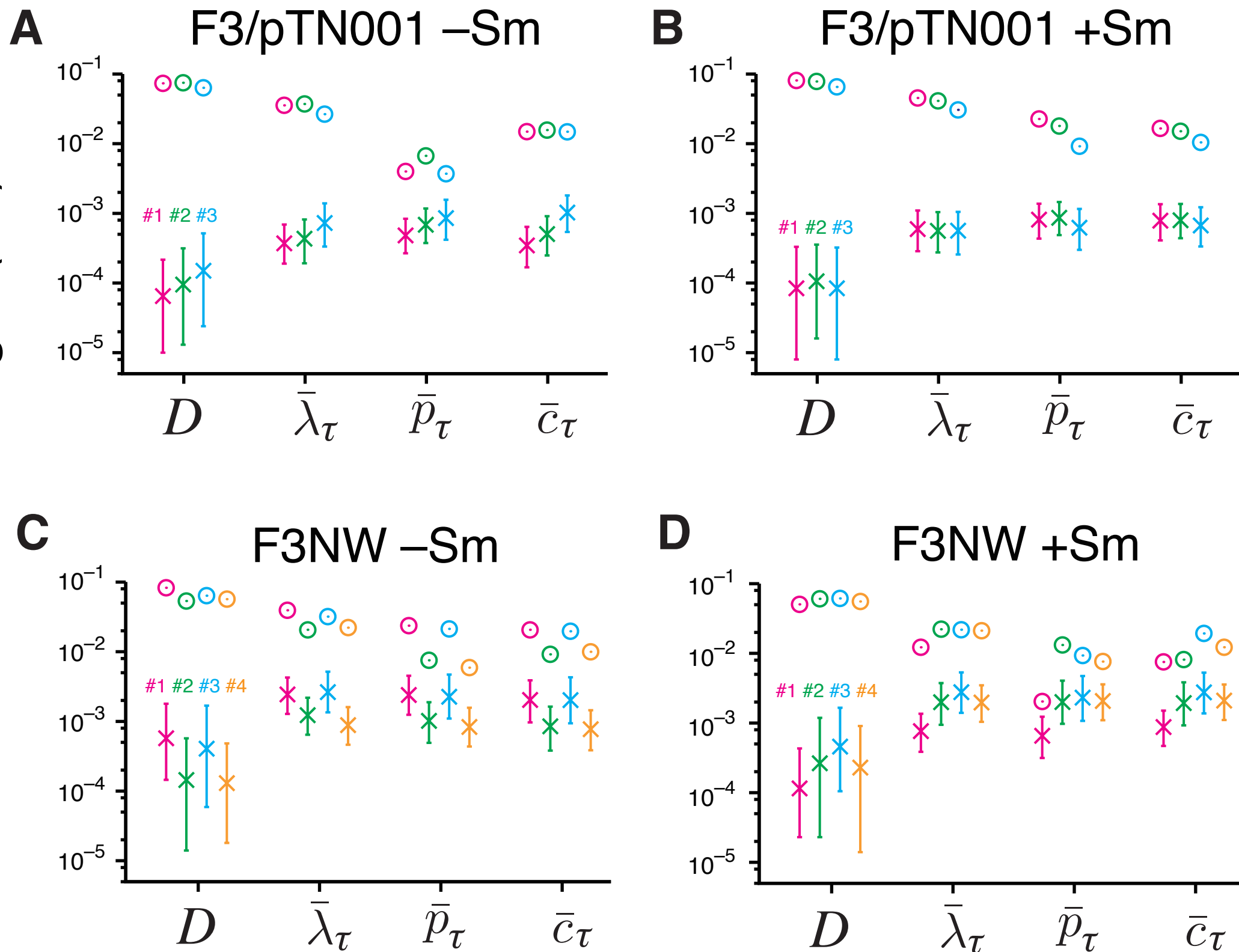

Supplement: S10 Fig — The combination of D and x (x = D, λ¯τ, p¯τ and c¯τ) was randomly shuffled over the lineages in each measurement, and selection strength S[x] was computed for each realization of the shuffle (Sshuffle[x]). The shuffle was repeated 10000 times, and the median and the 95% confidence interval were computed (shown as cross points with error bars) for all the independent measurements. A-D. 95% CI of Sshuffle[x] and original selection strength value Sori[x] (same as those calculated in Figs 6 and 7 in Main Text) were compared for all the independent measurements and phenotypes D, λ¯τ, p¯τ and c¯τ. The strain (F3/pTN001 or F3NW) and drug condition (−Sm or +Sm) are shown in each panel. S[x] is grouped by phenotype, and different color indicates different experiment. (PDF) [file pgen.1006653.s014.pdf]
