# Supplementary material for: Inferring fitness landscapes and selection on phenotypic states from single-cell genealogical data
Source: PLoS Genet. 2017 Mar 7;13(3):e1006653. doi: 10.1371/journal.pgen.1006653 (PMC5360348; doi:10.1371/journal.pgen.1006653)

A

	− SM					+ SM				
Time (min)	#1	#2	#3	#4	Ave.±SD	#1	#2	#3	#4	Ave.±SD
100	0.03	−0.17	−0.24	−0.07	−0.11±0.10	0.20	0.20	0.24	0.00	0.16±0.09
200	−0.17	−0.25	−0.09	−0.24	−0.19±0.07	−0.20	−0.12	−0.26	−0.10	−0.17±0.06
300	−0.32	−0.30	−0.33	−0.26	−0.30±0.03	−0.24	−0.22	−0.22	−0.30	−0.24±0.04

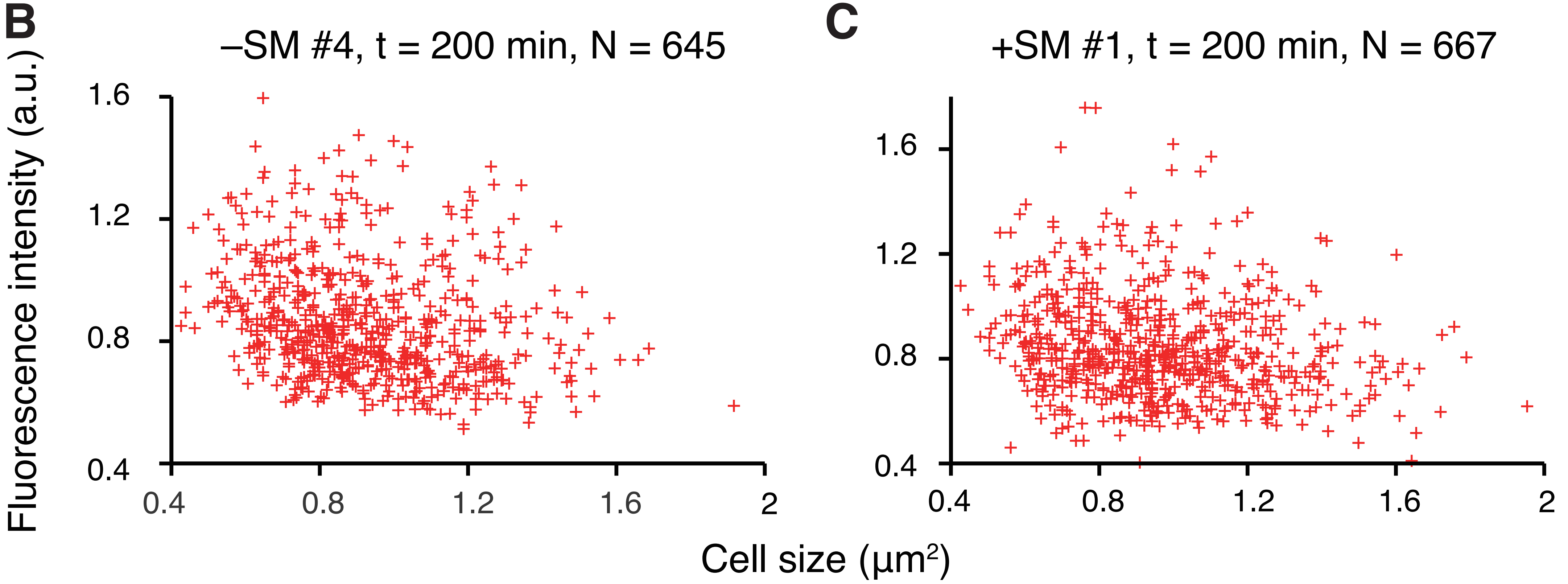

Supplement: S5 Fig — A. Correlation coefficients between fluorescence intensity c and cell size v in the data of F3NW for −Sm and +Sm conditions at three different time points, 100, 200 and 300 min. Averages and standard deviations of the correlation coefficients among three independent measurements for each drug condition are also shown. Those data suggest that c and v are almost uncorrelated. Typical scatter plots of c vs v are shown in B and C. B. Scatter plot of c vs v for the measurement #4 with −Sm condition for F3NW at 200 min. C. Scatter plot of c vs v for the measurement #1 with +Sm condition for F3NW at 200 min. (PDF) [file pgen.1006653.s009.pdf]
