# Supplementary material for: Inferring fitness landscapes and selection on phenotypic states from single-cell genealogical data
Source: PLoS Genet. 2017 Mar 7;13(3):e1006653. doi: 10.1371/journal.pgen.1006653 (PMC5360348; doi:10.1371/journal.pgen.1006653)
Supplement: S8 Fig — The chronological and retrospective probability distributions and the fitness landscapes for the three phenotypes of F3NW are shown. A. Time-averaged elongation rate, −Sm. B. Time-averaged elongation rate, +Sm. C. Time-averaged protein production rate, −Sm. D. Time-averaged protein production rate, +Sm. E. Time-averaged protein concentration, −Sm. F. Time-averaged protein concentration, +Sm. (PDF) [file pgen.1006653.s012.pdf]

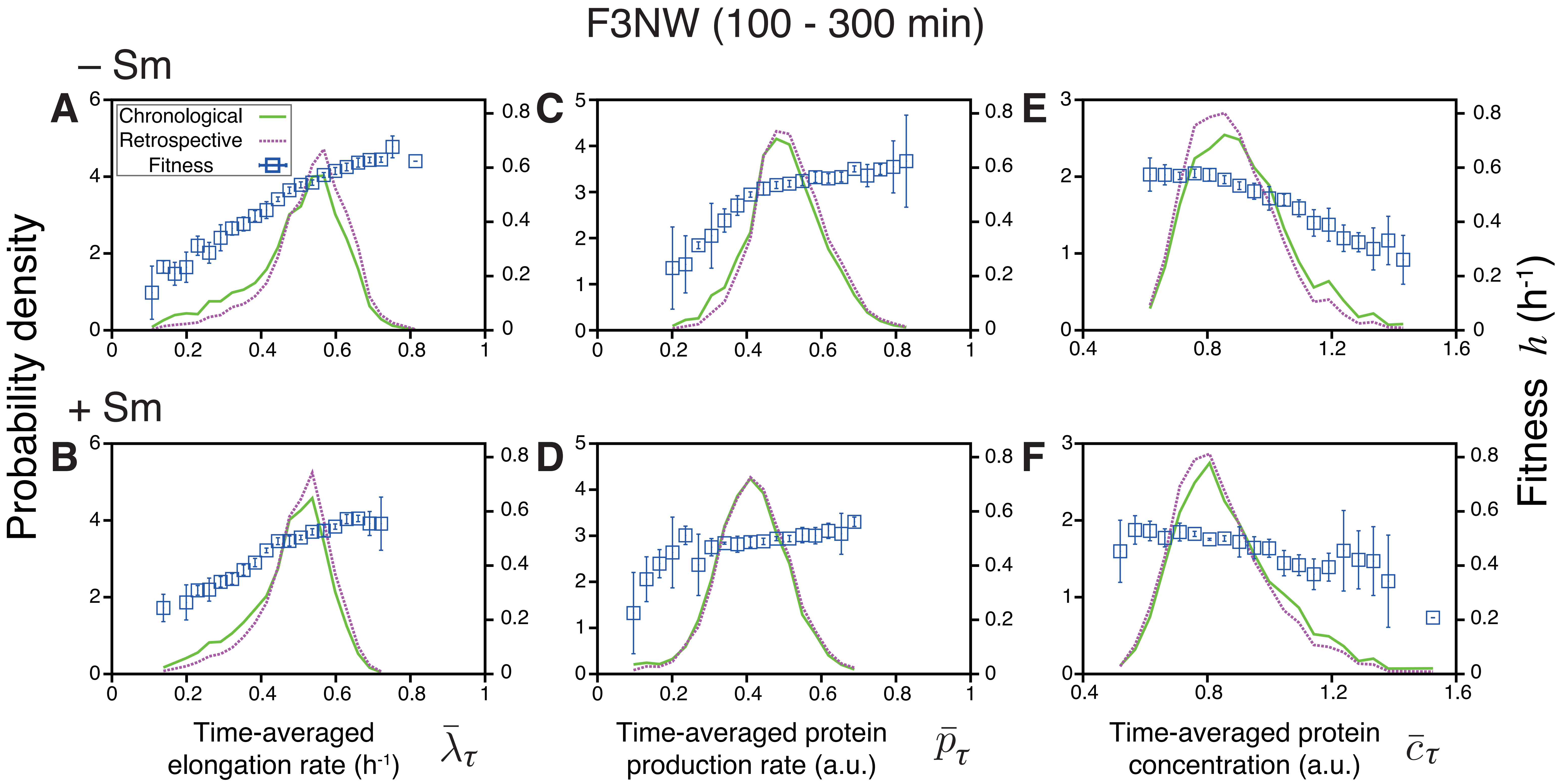
F3NW (100 - 300 min)
– Sm
+ Sm
A
B
C
D
E
F
Chronological
Retrospective
Fitness
Probability density
Fitness h (h^{-1})
Time-averaged elongation rate (h^{-1}) $\bar{\lambda}_\tau$
Time-averaged protein production rate (a.u.) $\bar{p}_\tau$
Time-averaged protein concentration (a.u.) $\bar{c}_\tau$
