# Supplementary material for: Inferring fitness landscapes and selection on phenotypic states from single-cell genealogical data
Source: PLoS Genet. 2017 Mar 7;13(3):e1006653. doi: 10.1371/journal.pgen.1006653 (PMC5360348; doi:10.1371/journal.pgen.1006653)
Supplement: S13 Fig — Results are of three replicates for each drug conditions (− Sm or + Sm). (PDF) [file pgen.1006653.s017.pdf]

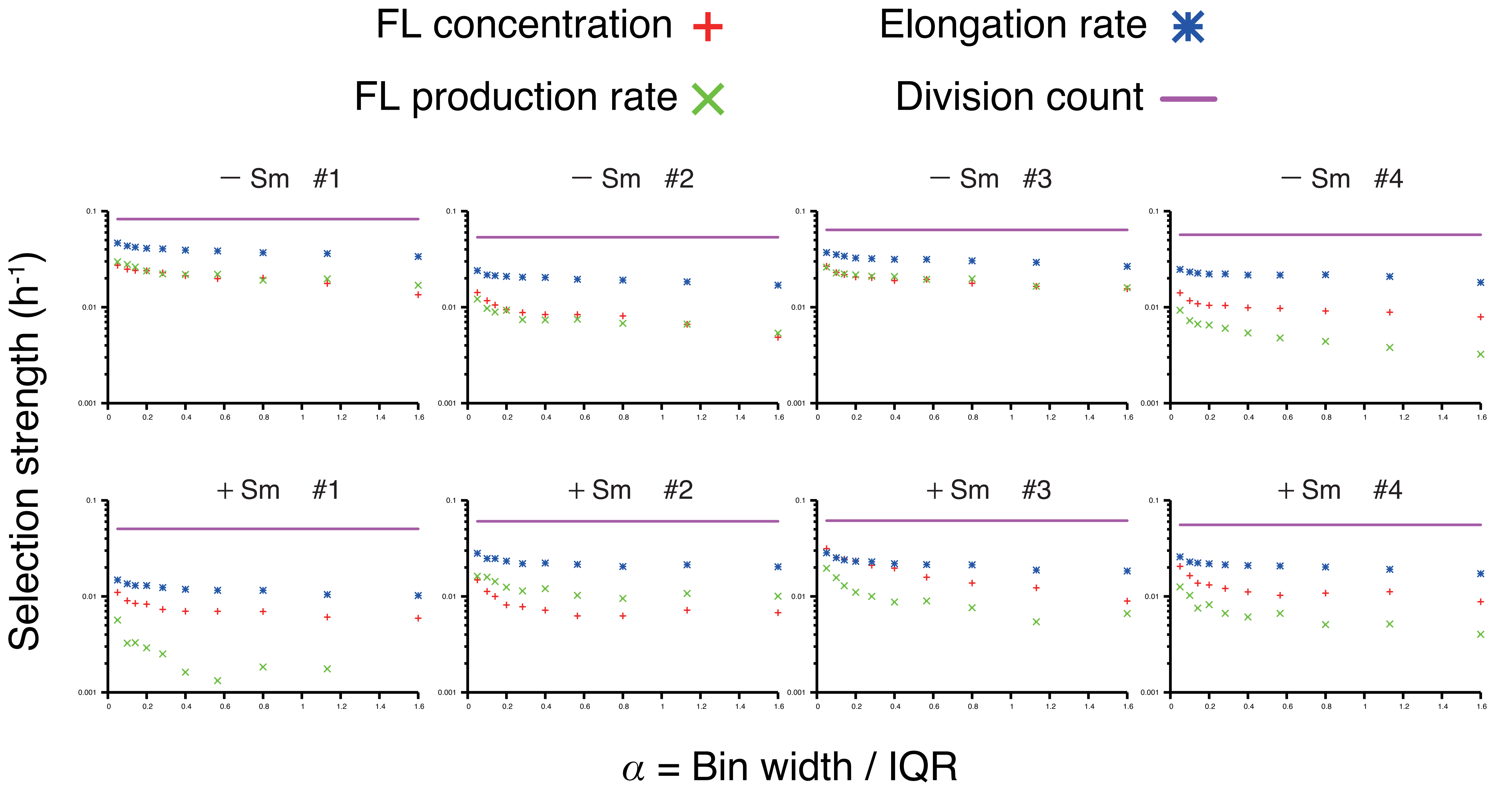
FL concentration
Elongation rate
FL production rate
Division count
− Sm #1
− Sm #2
− Sm #3
− Sm #4
+ Sm #1
+ Sm #2
+ Sm #3
+ Sm #4
Selection strength (h-1)
α = Bin width / IQR
